# Experimental dataset investigating the effect of temperature in the presence or absence of catalysts on the pyrolysis of plantain and yam peels for bio-oil production

**DOI:** 10.1016/j.dib.2020.105804

**Published:** 2020-06-03

**Authors:** Vincent E. Efeovbokhan, Damilola Akinneye, Augustine O. Ayeni, James A. Omoleye, Oladotun Bolade, Babalola A. Oni

**Affiliations:** a Department of Chemical Engineering, Covenant University, P.M.B 1023, Ota, Ogun State, Nigeria; b Department of Chemistry, Covenant University, P.M.B 1023, Ota, Ogun State, Nigeria

**Keywords:** Pyrolysis, Plantain peel, Yam peel, Zeolite-y, Heterogeneous catalysis, Bio-oil

## Abstract

More than 1.3 billion tons, a third of the total food produced, is wasted annually, and it has been predicted to increase in the coming years. Food waste significantly contributes to greenhouse gas (GHG) emissions resulting in the release of about 3.3 billion tonnes of CO_2_ into the environment yearly. Hence this large amount of wastes, with adverse environmental effects, needs to be appropriately managed. New technologies such as Anaerobic digestion, fermentation, and gasification are being used to produce renewable energy, which in turn reduces the increasing level of food wastes in the environment. Pyrolysis of biomass materials or food wastes produces high-value energy products or bio-oil that can possibly replace non-renewable fossil fuels when it is upgraded.

In this study, pyrolysis (thermal treatment in the absence of oxygen) of plantain and yam peels to produce bio-oil, was investigated. The pyrolysis conditions, wide temperature ranges at an interval of 100 °C (200–700 °C), absence of a catalyst (AOC), the use of zeolite –Y catalyst using two separate heterogeneous catalysis procedures were imposed and used to produced bio-oil. In the first procedure, the pyrolysis gases were allowed to rise through a zeolite-Y catalyst bed (HTC). And in the second procedure, the plantain or yam peel feedstock was first mixed uniformly with the zeolite-Y catalyst before pyrolysis (HMC). The GC–MS machine was used to analyze or characterize the obtained bio-oil while proximate analysis and XRF machine were used to characterize the plantain and yam peels feed. The residue, biochar, from the pyrolysis process, was also characterized using the XRF machine.

**Specifications Table**

**Table:** 

**Subject**	Chemical Engineering
**Specific subject area**	Waste conversion to bio-products
**Type of data**	Tables and figures
**How data were acquired**	Measurement, Proximate, X-ray fluorescence, and Gas Chromatography-Mass Spectroscopy analyses.
**Data format**	Raw
**Parameters for data collection**	The parameters considered were: yield and composition of the bio-oil and biochar obtained at the different temperatures, in the absence of a catalyst, and the presence of zeolite-Y catalyst using two heterogeneous catalysis procedures, HTC and HMC respectively.
**Description of data collection**	Data collected were through proximate analysis of the raw feed, X-ray fluorescence to characterize the raw feed and the residual biochar, and Gas Chromatography-Mass Spectroscopy analyses to characterize the bio-oil product at the different temperature ranges used during the pyrolysis process.
**Data source location**	Department of Chemical Engineering, Covenant University, Nigeria
**Data accessibility**	Data is with this article

**Value of the Data**

• Pyrolysis process makes it possible to convert food waste materials and, in this case, plantain peel, to bio-oil useful for renewable energy generation, or the production of valuable biochemicals.
• The temperature dataset provides significant insight into the required optimum reaction temperature for optimal bio-oil production during the pyrolysis of plantain and yam peels.
• The dataset obtained from the pyrolysis process without the use of a catalyst, in comparison with the two heterogeneous catalysis processes (HTC and HMC), will guide researchers with the best reaction conditions during the pyrolysis of plantain and yam peels.
• The GC–MS dataset from the characterization of the obtained bio-oil from plantain and yam peels will provide researchers with the biochemical constituents and other possible applications of the bio-oil.

## Data

Kitchen waste refers to left-over organic matter from eateries, hotels, and homes [1]**.** They are usually rich in nutrients containing a high amount of carbohydrates, lipids, and other organic molecules [2]. According to the Food and Agriculture Organization (FAO), a whopping 1.3 billion tons, a third of the produced food in the world, is wasted annually. Usually, food wastes are disposed of by landfilling, with only a little percentage used to produce feeds, compost, and biogas. The dataset presented in this report article shows the effect of variation of temperature with or without catalysis on the yield and chemical composition of bio-oil.

**Fig. 1:**
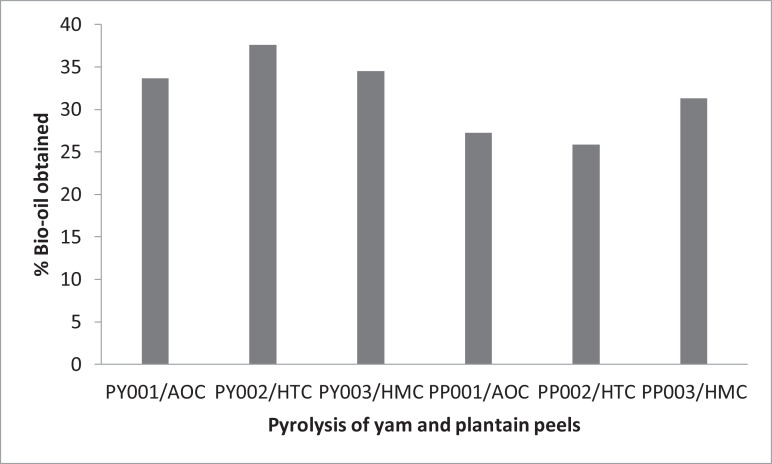
Total percentage yield of bio-oil from the different pyrolysis runs.

Bio-oils obtained from biomass, including food and agricultural wastes, have been reported as being a potential replacement of fossil fuels for renewable energy generation and the production of value-adding biochemicals [3], [4], [5]. It is, therefore, essential to investigate the feasibility of obtaining and characterizing bio-oils from plantain and yam peel, which are organic kitchen wastes, under varying conditions of temperatures and catalysis. The dataset presented in this article shows the yields, full characterization of the raw peels of plantain and yam feed, biochar, and the bio-oils obtained under the different pyrolysis conditions. Table 1 shows the proximate analysis of both the yam and plantain peels. Table 2 shows the XRF analysis of plantain peel, yam peel, and biochars, which are the by-products of the pyrolysis of the plantain and yam peels. Table 3, Table 4, Table 5, Table 6 show the bio-oil yields at the indicated temperature ranges for the pyrolysis without catalyst (AOC) and heterogeneous catalysis (HTC) using zeolite-Y as the catalyst. And the bio-oil yields were calculated using Eq. (1). The temperature ranges were (250–350, 350–450, 450–550, 550–650 °C) and (200–300, 300–400, 400–500, 500–600, 600–700 °C) for the pyrolysis without catalyst (AOC) and heterogeneous catalysis (HMC) using zeolite-Y as the catalyst, respectively. The two temperature ranges were the same for the heterogeneous pyrolysis (HTC) of the yam peel. Table 7 gives the total percentage of each of the pyrolysis products for any given experimental run. And the yields of the biochar and the non-condensable products calculated using Eqs. (2) and 3, respectively. Table 8, Table 9, Table 10, 11–13, 14–17, and 18–20, give the bio-chemical compositions of the obtained bio-oils at each of the above-stated pyrolysis conditions, (with and without catalysis, and the different temperature ranges). The bio-chemical compositions of the bio-oil were analyzed, using the Gas Chromatography-Mass Spectroscopy (GC–MS) machine. The chromatograms for the various bio-oil samples obtained, at the different pyrolysis conditions, are presented in the supplementary or chromatography file submitted to Data-in-Brief. The chromatograms (Figures 1–18) depict the biochemical compositions of the analyzed bio-oil samples, and they are interpreted and shown in Table 8, Table 9, Table 10, Table 11, Table 12, Table 13, Table 14, Table 15, Table 16, Table 17, Table 18, Table 19, Table 20.

**Table 1:** Proximate analyses of plantain and yam peel feedstock.

Analysis	Plantain Peel	Yam Peel
Moisture Content (%)	8.8	7.5
Ash Content (%)	4.6	7.5
Protein Content (%)	21.9	2.4
Fat Content (%)	8.4	4.2
Crude fibre Content (%)	21.8	9
Carbohydrate Content (%)	34.6	69.4

**Table 2:** XRF Analysis of raw plantain peel, yam peel, and their biochars.

Detected compounds	% composition in plantain peel	% composition in plantain peel	% composition in plantain peel biochar	% composition in yam peel biochar
SiO_2_	0.35	0.30	0.18	0.21
SO_3_	0.45	0.26	Nd	Nd
P_2_O_5_	Nd	Nd	0.04	0.90
Cl	2.05	2.38	1.77	0.95
CaO	Nd	Nd	Nd	Nd
TiO_2_	0.38	0.32	0.05	0.25
Fe_2_O_3_	0.53	2.28	0.76	2.1
CuO	0.12	0.44	0.05	0.11
ZnO	0.07	Nd	0.06	0.08
SrO	Nd	Nd	Nd	Nd
Nb_2_O_5_	0.03	Nd	0.05	Nd
T_2_O_5_	Nd	Nd	Nd	0.14
CdO	Nd	Nd	Nd	Nd
HfO_2_	Nd	Nd	Nd	Nd
PbO	Nd	Nd	Nd	Nd
Br	Nd	0.19	0.03	Nd
RuO_2_	1.30	4.50	0.59	0.64
Dy_2_O_3_	0.52	1.60	0.34	0.54
Organic matter	93.40	86.00	95.9	93.66

**Table 3:** Yields of Bio-oil from the pyrolysis of plantain and yam peels without catalyst (AOC) at the different temperature ranges.

Temperature Range ( °C)	Percentage Yield	Yam Percentage Yield
250–350	4.9	6.4
350–450	14.9	6.9
450–550	4.7	8.4
550–650	2.7	11.9

**Table 4:** Yields of Bio-oil from the pyrolysis of plantain peel with heterogeneous catalysis (HTC) at the different temperature ranges.

Temperature Range (°C)	Percentage Yield
250 – 350	4.3
350 – 450	6.9
450 – 550	7.2
550 – 650	7.5

**Table 5:** Yields of Bio-oil from the pyrolysis of yam peel with heterogeneous catalysis (HTC) at the different temperature ranges.

Temperature Range ( °C)	Percentage Yield from yam peel
200–300	4.1
300–400	6.3
400–500	18.5
500–600	5.9
600–700	2.7

**Table 6:** Yields of Bio-oil from the pyrolysis of plantain and yam peels with heterogeneous catalysis (HMC) at the different temperature ranges.

Temperature Range ( °C)	Percentage Yield from Plantain peel	Percentage Yield from yam peel
200–300	7.5	5.7
300–400	6.3	6.8
400–500	8.2	8.9
500–600	8	9.9
600–700	1.3	3.3

**Table 7:** Pyrolysis products distribution from the various experimental runs.

Run	Bio-oil (%)	Char (%)	Non-Condensables (%)
Y001/AOC	33.67	28.467	37.866
Y002C/HTC	37.6	30.267	32.133
Y003C/HMC	34.53	21.6	43.867
P001/AOC	27.27	35.8	36.933
P002/HTC	25.87	31.733	42.4
P003C/HMC	31.33	24.2	44.467

PY001/AOC – pyrolysis of yam peel without catalyst

PY002/HTC – pyrolysis of yam peel with heterogeneous catalysis (HTC)

PY003/HMC – pyrolysis of yam peel with heterogeneous catalysis (HMC)

PP001/AOC – pyrolysis of plantain peel without catalyst

PP002/HTC – pyrolysis of plantain peel with heterogeneous catalysis (HTC)

PP003/HMC – pyrolysis of plantain peel with heterogeneous catalysis (HMC).

**Table 8:** GC–MS results of bio-oil obtained from plantain peel without catalyst (AOC) at 350–450 °C.

Peak	Retention Time	Area Percentage	IUPAC Nomenclature
1.	3.1271–3.1786	1.4258	3-Aminopyridine
2.	3.3045	0.77	Cyclopentanone, 2-methyl-
3.	3.3731	0.996	dl-Erythro-O-methylthreonine
4.	3.4304	2.7564	2-Hexanol, 2,5-dimethyl-, (*S*)-
5.	3.5677–3.8595	3.9876	2-Furanmethanol
6.	4.0369	3.3905	2-Cyclopenten-1-one, 2-methyl-
7.	4.2028	0.2374	Hexanoic acid
8.	4.363	0.2445	Pyridine, 3,5-dimethyl-
9.	4.4203	0.5496	Cyclohexene, 1,2-dimethyl-
10.	4.4889	2.3506	Butyrolactone
11.	4.6549	0.2624	2-Pyridinecarboxylic acid, 6-methyl-
12.	4.7865	2.5153	2-Cyclopenten-1-one, 3-methyl-
13.	4.9524	4.1246	1-Ethylcyclopentene
14.	5.0039–5.1813	9.1488	Phenol
15.	5.29	1.2068	1,2-Dithiolane-1-oxide
16.	5.4674	0.914	Silane, hexyl-
17.	5.5017	0.3318	Butanal, 3-methyl-, oxime
18.	5.5875	1.4401	Pyrazine, methyl-
19.	5.7706	6.7513	2-Cyclopenten-1-one, 2,3-dimethyl-
20.	5.9194	1.2399	Phenol, 2-methyl-
21.	5.9824	0.353	2-Pyrrolidinone, 1-methyl-
22.	6.0625	1.5404	Phenol, 2-methyl-
23.	6.1883–6.3085	3.7875	p-Cresol
24.	6.4058	0.7644	1H-Pyrrole-2-carboxaldehyde, 1-methyl-
25.	6.5088	1.8054	Hexane, 2,4-dimethyl-
26.	6.6461	1.3608	Sulfurous acid, butyl 2-ethylhexyl ester
27.	6.8063	2.4219	3-Piperidinone, 1,6-dimethyl-
28.	6.9379	1.4011	2-Furancarboxylic acid, hydrazide
29.	7.0581	2.4507	Phenol, 4-amino-
30.	7.1725	2.0683	4(1H)-Pyridinone, 2,3-dihydro-1-methyl-
31.	7.2812	0.8078	Phenol, 2,5-dimethyl-
32.	7.3556	0.5552	Pyrazine, 3,5‑diethyl-2-methyl-
33.	7.4529	0.3425	3-Buten-2-one, 4-(1-aziridinyl)-
34.	7.5044	0.6484	Phenol, 4-ethyl-
35.	7.533	0.5956	Phenol, 2,3-dimethyl-
36.	7.6646	0.9852	3-Dimethylaminoacrylonitrile
37.	7.7333	0.7659	Ethanone, 1-(4-fluorophenyl)-
38.	7.8477	0.6243	4-Pyridinemethanol
39.	7.8992	0.2322	2-Furanmethanol, tetrahydro-5-methyl-
40.	7.985	0.8648	Fumaric acid, 2-ethylphenyl isohexyl ester
41.	8.1395	5.3891	1,4:3,6-Dianhydro-.alpha.-d-glucopyranose
42.	8.357	1.1478	Diethyl selenide
43.	8.5744	0.3373	2,4-Hexadiene, 2,5-dimethyl-
44.	8.6259	0.3398	1H-Imidazole, 2,4,5-trimethyl-
45.	8.7175	1.141	Picolinamide
46.	8.7861	0.594	Pyrrolidine, 1-(2-methyl-1-propenyl)-
47.	8.8262	0.3569	4-Acetylocta-1,2-diene
48.	8.9234	0.4907	Nicotinyl alcohol
49.	8.9807	0.2341	Phenol, 3-amino-
50.	9.1523	1.2146	Indole
51.	9.2038	0.9453	Pyrrolidine, 1-(1-butenyl)-
52.	9.4098	0.4041	7-Azabicyclo[4.1.0]heptane, 2-methyl-5-(1-methylethyl)-
53.	9.5071	0.4073	1-Methoxy-1,4-cyclohexadiene
54.	9.6387	0.2287	1-Azabicyclo[3.2.1]octane, 6-methyl-, endo-
55.	9.7302	0.3647	trans-4a-Methyl-decahydronaphthalene
56.	9.7989	0.4211	Phenol, 2,6-dimethoxy-
57.	9.8733	0.2651	2,3,4-Trimethylpyrrole
58.	9.9191	0.3242	5-t-Butyl-hexa-3,5‑dien-2-one
59.	10.0106–10.1994	0.5053	2,4-Imidazolidinedione, 5-methyl-
60.	10.1994	0.2055	1-Methoxy-1,4-cyclohexadiene
61.	10.806	0.6757	5-Ethylhydantoin
62.	10.8403	0.6598	1H-Phosphole, 2,5-dihydro-1-methyl-
63.	11.1607	0.6617	Mexiletine acetate
64.	11.218	0.1731	Isoetharine
65.	11.4239	0.2119	t-Butylhydroquinone
66.	11.7329	0.9313	1-Butyl-3,4-dihydroxy-pyrrolidine-2,5-dione
67.	11.813	0.4639	1,3-Oxathiolane, 2-ethyl-2-methyl-
68.	11.876	0.2509	Pyrazine, 5‑butyl‑2,3-dimethyl-
69.	11.9618	0.1832	Benzenemethanol, .alpha.-ethynyl-
70.	12.7171	0.2988	Benzenamine, 3,5-dimethyl-4-[2-*oxo*-2-(1-*pyrrolidinyl*)*ethoxy*]-
71.	13.0204	0.3857	Cyclohexanecarbonitrile, 1-(1-piperidinyl)-
72.	13.3923	0.2953	N-[6-[N-A*ziridyl*]−3-aza-3-hexenyl]morpholine
73.	13.4896	0.5758	Cyclopentanecarboxamide, N,N-dimethyl-
74.	13.5983	0.5218	3,4-Difluorobenzyl alcohol, methyl ether
75.	14.016	0.4825	2-Amino-4,5-dimethylthiazole
76.	14.1362	0.4994	Ethanone, 1-(2,2-dimethylcyclopentyl)-
77.	14.8743	0.1704	Glycyl-l-proline
78.	15.1261	0.7781	2-Hydroxy-3,5,5-trimethyl-cyclohex-2-enone
79.	15.8985	0.2078	Pyrrolo[1,2-*a*]pyrazine-1,4‑dione, hexahydro-3-(2-methylpropyl)-
80.	16.0244	2.6051	5,10-Diethoxy-2,3,7,8-tetrahydro-1H,6H-dipyrrolo[1,2-*a*:1′,2′-*d*]pyrazine
81.	16.9514	0.1838	Phthalimide, N-isopropyl-
82.	17.5522	0.5081	Octadec-9-enoic acid
83.	17.5922	0.3906	6-Octadecenoic acid
84.	17.7296	0.2002	Octadecanoic acid
85.	21.672	0.5307	Z-17-Nonadecen-1-ol acetate

**Table 9:** GC–MS results of bio-oil obtained from plantain peel without catalyst (AOC) at 450–550 °C.

Peak	Retention Time	Area Percentage	IUPAC Nomenclature
1	3.1211	0.2357	Pyrimidine, 5-methyl-
2	3.1669	1.7354	N,N-Dimethylaminoethanol
3	3.3042	0.3734	Cyclopentanone, 2-methyl-
4	3.3672	0.6818	Butane, 1-ethoxy-
5	3.4244	3.7099	2-Hexanol, 2,5-dimethyl-, (*S*)-
6	3.556	1.7328	Butanoic acid
7	3.7219–3.8535	2.2784	2-Furanmethanol
9	3.9394	0.6356	Methyl 6-methyl heptanoate
10	4.0652	3.036	2-Cyclopenten-1-one, 2-methyl-
11	4.0881	2.1236	1,4-Pentadiene, 2,3,3-trimethyl-
12	4.1968	0.2657	1-Penten-3-ol, 3-methyl-
13	4.2655	0.4263	Propanedioic acid, propyl-
14	4.3628	0.2494	Pyridine, 3,5-dimethyl-
15	4.4028	0.2267	Piperazine, 2-methyl-
16	4.4829	1.9733	Butyrolactone
17	4.6546	0.2211	2-Pyridinecarboxylic acid, 6-methyl-
18	4.7805–4.9407	3.7735	2-Cyclopenten-1-one, 3-methyl-
20	4.9922–5.1810	6.5586	Phenol
22	5.3069	0.6039	Ethanole, 2-[(2-*chloroethyl*)*thio*]-, acetate
23	5.5072	1.9498	Ethoxycarbonyl isothiocyanate
24	5.5816	1.269	Pyrazine, methyl-
25	5.7589	3.9833	2-Cyclopenten-1-one, 2,3-dimethyl-
26	5.9134	0.7849	Phenol, 2-methyl-
27	5.9707	0.4053	2-Pyrrolidinone, 1-methyl-
28	6.0565	1.0511	Phenol, 2-methyl-
29	6.1766	1.2406	2-Amino-4-methylpyrimidine
30	6.3083	0.5855	Phenol, 2-methyl-
32	6.5085	1.7836	2-Pyrrolidinone
33	6.5715	0.7634	Furan, 2,3,5-trimethyl-
34	6.6859	1.2165	1H-Imidazole, 1-(2-propenyl)-
35	6.8003	2.6506	3-Methyl-isoxazol-5(4H)-one
36	6.8862	0.4068	1H-Pyrazole, 1-methyl-3-vinyl-
37	6.9434	1.7694	Hexadecanamide
38	7.0693	3.4256	7-Octen-3-ol, 2,6-dimethyl-
40	7.218	1.3802	1-Hexadecen-3-ol, 3,5,11,15-tetramethyl-
41	7.2982	0.3258	Phenol, 2,5-dimethyl-
42	7.4526	0.4061	3-Buten-2-one, 4-(1-aziridinyl)-
43	7.4927	0.5165	1-Azabicyclo[2.2.2]octane, 3-methyl-
46	7.6758	1.5664	Hexahydroindole
47	7.7445	1.4645	3-Pyridinol, 2,6-dimethyl-
48	7.8532	1.4468	1,2-Cyclohexanedimethanol
49	7.9047	1.2069	2-(Chloromethyl)tetrahydropyran
50	7.9505	0.7762	7-[2-T*ertahydropyranyloxy*]−1-heptyne
51	7.9962	1.7	1H-Pyrrole-2,5‑dione, 1-(hydroxymethyl)-
52	8.0706	0.184	2(1H)-Pyridinone, 3-methyl-
53	8.145	3.5442	1,4:3,6-Dianhydro-.alpha.-d-glucopyranose
54	8.1908	1.1197	2-Methyl-2-pentyl methylphosphonofluoridate
55	8.3624	1.712	2(1H)-Pyridinone, 1,5-dimethyl-
56	8.4711	0.6972	2-Ethyl-6-methyl-pyridin-3-ol
57	8.5799	0.2859	2,5-Furandione, 3-(1,1-dimethylethyl)-
58	8.6257	0.2319	Phenol, 2-(dimethylamino)-
59	8.7115	0.9618	Picolinamide
60	8.7859	0.3929	1-(4-Fluorophenyl)−2-methyl-2-propanol
61	8.8374	0.4186	Benzenamine, 3‑methoxy-
62	8.9289	0.5277	3,4-Dimethyl-3-pyrrolin-2-one
63	8.9919	0.3438	4-Pyridinemethanol
64	9.0319	0.2277	8-Azabicyclo[3.2.1]oct‑2-ene
65	9.1063	0.4004	Phenol, 4-amino-
66	9.1578	0.6429	Benzonitrile, 3-methyl-
67	9.215	0.9347	Pyrrolidine, 1-(1-butenyl)-
68	9.3752	0.2243	Pentane, 3-(2,2-dichloro-3-methylcyclopropyl)-
69	9.4096	0.3124	1-Methoxy-1,4-cyclohexadiene
70	9.5068	0.3326	Benzenamine, 3‑methoxy-
71	9.6384	0.2875	1-Azabicyclo[3.2.1]octane, 6-methyl-, endo-
73	9.7987	0.3098	Benzene, 1‑methoxy-2-(methylthio)-
74	10.0676	0.7511	2,4-Imidazolidinedione, 5-methyl-
75	10.1191	0.4678	Creosol
76	10.2049	0.8601	Imidazole, 1,4,5-trimethyl-
77	10.3308	0.2518	1-Vinyl-2-hydroxymethylimidazole
80	10.7027	0.2276	1H-Isoindole-1,3(2H)‑dione, 2-methyl-
81	10.8458	0.9022	5-Ethylhydantoin
82	11.1605	0.478	N-Isobutyl‑sec-butylamine
83	11.2463	1.2247	3-Morpholinopropyl 4‑hydroxy-3-methoxybenzoate
84	11.4237	0.2624	Benzenethiol, 4-(1,1-dimethylethyl)-
85	11.7498	0.7623	Acetamide, N-(4-hydroxycyclohexyl)-, cis-
86	11.8185	0.4394	Methyl tetra-p-nitrobenzate-.beta.-O-galactopyranoside
87	12.2763	0.7384	4,5,6,7-Tetrahydrobenzo[*c*]thiophene-1-carboxylic acid, (2-morpholin-4-ylethyl)amide
88	12.6081	0.212	Phenol, 3-methyl-6-propyl-
89	13.4893	0.228	N-[6-[N-A*ziridyl*]−3-aza-3-hexenyl]morpholine
91	14.0157	0.4252	2-Acetylamino-3-cyano-propionic acid
92	14.1359	0.373	Ethanone, 1-(2,2-dimethylcyclopentyl)-
93	14.3476	0.2928	Pyrrolo[1,2-*a*]pyrazine-1,4‑dione, hexahydro-
94	15.1258	0.8114	Pyrrolo[1,2-*a*]pyrazine-1,4‑dione, hexahydro-3-(2-methylpropyl)-
97	17.5519	0.382	6-Octadecenoic acid
98	17.5863	0.2795	9-Octadecenoic acid, (E)-
99	20.4015	0.2762	Octadecanedioic acid
100	21.6717	0.3898	1,2-Bis(trimethylsilyl)benzene

**Table 10:** GC–MS results of bio-oil from plantain peel without catalyst (AOC) at 550–650 °C.

Peak	Retention Time	Area Percentage	IUPAC Nomenclature
1	3.1268,4.9693, 51,925	7.983	Phenol
2	3.2012	1.3477	N,N-Dimethylaminoethanol
3	3.4473	3.5293	2-Hydroxy-2,6-dimethyl‑hept-6-en-3-one
4	3.6132	3.8054	Butanoic acid
5	3.7505–38,707	1.8988	2-Furanmethanol
7	3.9565	0.8158	Propanal, 3‑methoxy-
8	4.0366	1.4251	2-Cyclopenten-1-one, 2-methyl-
9	4.1053	4.7529	Cyclobutene, 1,2,3,4-tetramethyl-
10	4.214	0.1813	1-Penten-3-ol, 3-methyl-
11	4.317	1.0146	Pentanoic acid
12	4.3628	0.3812	Pyridine, 3,5-dimethyl-
13	4.42	0.2972	1,2-Epoxynonane
14	4.4944	1.5447	Butyrolactone
15	4.5287	0.3777	Propanamide, 2-methyl-
16	4.6546	0.2024	2-Acetyl-4-methylpyridine
17	4.7919	2.0571	2-Cyclopenten-1-one, 3-methyl-
19	5.1353	0.2864	2-Cyclopenten-1-one, 3,4-dimethyl-
21	5.3355	0.7439	Octanoic acid
22	5.5358	2.6799	Butanal, 3-methyl-, oxime
23	5.6044	1.4312	Pyrazine, methyl-
24	5.7589	3.669	2-Cyclopenten-1-one, 2,3-dimethyl-
25	5.9134	0.6839	Phenol, 2-methyl-
26	5.9821	0.6059	2-Pyrrolidinone, 1-methyl-
27	6.0679	1.0915	Phenol, 2-methyl-
28	6.1938	1.6754	2-Amino-4-methylpyrimidine
29	6.3311	0.5769	p-Cresol
30	6.4399	2.2297	1-Pentanol, 2,2-dimethyl-
31	6.5085–6.5429	2.1058	2-Pyrrolidinone
33	6.6001	0.4974	1,1-Dimethyl-4-methylenecyclohexane
34	6.7145	2.1413	dl-Mevalonic acid lactone
35	6.8576	3.6281	3-Piperidinone, 1,6-dimethyl-
36	6.972	0.86	Mannosamine
37	7.0063	0.6455	Pentaleno[1,2-*b*]oxirene, octahydro-, (1a.alpha.,1b.alpha.,4a.beta.,5a.alpha.)-
38	7.1093	1.9984	1H-Imidazole, 2-ethyl-4-methyl-
39	7.1723	1.5287	3-Buten-2-one, 4-(1-aziridinyl)-
40	7.2467	2.0688	1-Octen-3-ol, methyl ether
41	7.321	0.5543	Phenol, 2,5-dimethyl-
42	7.487	0.8777	9-Decen-2-ol
43	7.5614	0.5184	Phenol, 2,3-dimethyl-
44	7.6358–7.6873	1.1900	3-Dimethylaminoacrylonitrile
46	7.7902	0.8443	Ethanone, 1-(4-fluorophenyl)-
47	7.8704	0.7913	4-Pyridinemethanol
48	7.9276	0.5332	3-Buten-2-ol, 2,3-dimethyl-
49	8.0134	1.8067	2H-Pyran, tetrahydro-2-(12-pentadecynyloxy)-
50	8.1679	4.5668	1,4:3,6-Dianhydro-.alpha.-d-glucopyranose
51	8.3739	1.0336	2(1H)-Pyridinone, 3,6-dimethyl-
52	8.4254	0.7785	Benzenamine, 4-ethoxy-
53	8.4883	0.6939	2-Ethyl-6-methyl-pyridin-3-ol
54	8.5913	0.1804	Hexahydroindole
55	8.6371	0.3662	1,3,4,5,6,7-Hexahydro-2H-pyrindin-2-one
56	8.7229	0.5923	Picolinamide
57	8.7916	0.4857	Benzenemethanamine, 4-fluoro-
58	8.8603	0.4377	3-Pyridinol, 2,6-dimethyl-
60	9.0491	0.3224	Isoxazole-4-carbonitrile, 5-amino-3-methyl-
61	9.1635	0.7923	Benzonitrile, 2-methyl-
62	9.2322	0.9575	Pyrazine, methoxy‑
64	9.524	0.2715	9-Borabicyclo[3.3.1]nonane, 9-methyl-
65	9.6098	0.4552	3,4-dimethyl-1H-pyrrole-2-carboxaldehyde
66	9.6499	0.3776	1-Azabicyclo[2.2.2]octane, 4-methyl-
67	9.7643	0.5331	Creosol
68	9.8101	0.2093	Phenol, 2,6-dimethoxy-
69	9.936	0.1845	1H-Imidazole, 2,4,5-trimethyl-
70	10.0848	0.1733	Tricyclo[3.2.1.0(2,4)]octane-6-carboxamide, 6-methyl-3-oxa-
71	10.1248	0.3121	3-(.alpha.-Hydroxyethyl)-aniline
72	10.2221	0.5557	1-[3-A*minopropyl*]−2[1H]-pyridone
73	10.3365	0.5475	1-Vinyl-2-hydroxymethylimidazole
74	10.4967	0.3329	1H-Pyrazole, 1,3,4,5-tetramethyl-
75	10.6283	0.2992	Acetaldehyde, (3,3-dimethylcyclohexylidene)-, (Z)-
77	10.8629–11.1662	2.121	1H-Phosphole, 2,5-dihydro-1-methyl-
79	11.2463	0.7708	Diethylpropion
80	11.4294	0.3324	3,4-Dihydroxypropiophenone
81	11.6926	0.329	2-Methyl-4,6-quinolinediol
82	11.8357	0.1886	Tetraacetyl-d-xylonic nitrile
83	11.973	0.2328	2,5-Cyclohexadiene-1,4‑dione, 3‑hydroxy-2-methyl-5-(1-methylethyl)-
84	12.2877	0.355	Mexiletine acetate
85	12.6139	0.2563	Phenol, 3-methyl-6-propyl-
86	13.0316	0.6468	Cyclohexanecarbonitrile, 1-(1-piperidinyl)-
87	13.4893	0.2541	Nitro-l-arginine
89	14.0215	0.5315	4-Acetamido-4-ethyloctane
90	14.1531	0.5365	4-Undecene, 9-methyl-, (Z)-
92	14.5307	0.2683	4-Amino-6‑chloro-2-methoxypyrimidine
93	14.8798	0.1772	2-Hydroxy-3,5,5-trimethyl-cyclohex-2-enone
95	15.904	0.1964	Phenol, 3,5-dimethoxy-
97	17.5519	0.4072	9-Octadecenoic acid, (E)-
98	17.5863	0.2953	Z-9-Pentadecenol
99	20.4015	0.2747	2-Methylpiperidine-1-thiocarboxylic acid 2-[1-[2-*thiazolyl*]ethylidene]hydrazide
100	21.6717	0.3903	1,2-Benzisothiazol-3-amine tbdms

**Table 11:** GC–MS results of bio-oil from plantain peel with heterogeneous catalysis (HTC) at 350–450 °C.

Peak	Retention Time	Area Percentage	IUPAC Nomenclature
1	3.1325	2.6963	3-Aminopyridine
2	3.2985	0.8498	Cyclopentanone, 2-methyl-
3	3.3729	1.3141	4,5-Dimethyl-3-heptanol
4	3.4759	4.4594	Butanoic acid
5–8	3.5674–3.8478	4.9094	2-Furanmethanol
9	3.9737	0.2773	Hexanoic acid, 2-methyl-
10–11	4.0309–4.0767	8.6637	2-Cyclopenten-1-one, 2-methyl-
12	4.1911	1.2523	Chloro-methyl‑methoxy-amine
13	4.2598	0.3186	1,3-Benzenediamine
14	4.3742	0.5093	Pyridine, 2,4-dimethyl-
15	4.4715	1.9916	Butanoic acid, 4‑hydroxy-
16	4.5001	0.5577	1-Methylcycloheptene
17	4.6489	0.5237	2-Pyridinecarboxylic acid, 6-methyl-
18–19	4.7805–4.8663	3.7418	2-Cyclopenten-1-one, 3-methyl-
20–21	4.9464–4.9922	3.9725	2,4-Dimethylfuran
22	5.1524	5.5281	Phenol
23	5.2668	0.7714	1,3-Benzenediamine, 2-methyl-
24	5.4442	0.8795	Silane, diethyldimethyl-
25	5.5358	0.5489	Cyclopentane, 1,2-dimethyl-3-methylene-, trans-
26	5.5758	1.1402	2-Aminopyridine
27	5.7647	8.0174	2-Cyclopenten-1-one, 2,3-dimethyl-
28	5.9077	1.2935	Phenol, 2-methyl-
29	5.9706	0.2174	Imidosulfurous difluoride, methyl-
30	6.0507	0.685	Phenol, 2-methyl-
32	6.1709	0.8012	2-(1-Cyclopent-1-enyl-1-methylethyl)cyclopentanone
33	6.2968	1.4154	p-Cresol
34	6.4112	2.8538	2H-Pyran, 2-(7-dodecynyloxy)tetrahydro-
35	6.4971	0.632	1H-Imidazole, 2,4-dimethyl-
36	6.5428	0.3068	1H-Imidazole, 2-ethyl-4-methyl-
37	6.6172	1.0213	3-Cyclohexen-1-carboxaldehyde, 3-methyl-
38	6.663	0.2585	2-Pyridinamine, 3-methyl-
39	6.7088	0.2737	2H-Azepin-2-one, hexahydro-1-methyl-
40	6.7546	1.5263	3-Amino-4,5-dihydro-3H-[1,4′]bipyridinyl-2,6-dione
41	6.8404	0.4097	Maltol
42	6.8919	0.9168	9-Octadecenamide, (Z)-
43	6.9205	0.8763	2-Furancarboxylic acid, hydrazide
44	7.012	1.5099	2(1H)-Pyridinone, 3-methyl-
45	7.1551	1.8059	Cyclohexane, 1,1,3-trimethyl-
46	7.2638	0.5386	Phenol, 2,5-dimethyl-
49–50	7.487–7.5957	1.1022	1H-Imidazole, 2,4-dimethyl-
51	7.7158	0.2297	3-Fluorobenzoic acid, 4-cyanophenyl ester
52	7.836	0.9772	3,4,5-Trimethylpyrazole
53	7.8875	0.8181	2(3H)-Furanone, dihydro-5-pentyl-
54	7.9218	0.9454	3-Thiopheneacetonitrile
55	8.0992	3.3006	1,4:3,6-Dianhydro-.alpha.-d-glucopyranose
56	8.1793	0.6847	3,5-Octadien-2-one
57	8.3395	2.3405	3,4,5,6,7,8-Hexahydro-2H-chromene
60	8.6886	0.227	2-Hydrazinopyridine
64	9.0891	0.749	5-Fluoro-m-xylene
65	9.1406	1.4945	Bicyclo[2.2.1]heptane, 2,2,3-trimethyl-
66	9.2722	0.2494	2-Allyl-2-methyl-1,3-cyclopentanedione
67	9.3352	0.209	1H-Pyrazole, 1,3,5-trimethyl-
68	9.7872	0.4654	Phenol, 2,6-dimethoxy-
69	9.8559	0.5297	1-Methoxy-1,3-cyclohexadiene
70	9.9932	0.3195	2,4-Imidazolidinedione, 5-methyl-
71	10.1877	0.4461	3-Cyclohexene-1-carboxaldehyde, 1,3,4-trimethyl-
72	10.2793	0.3454	2-Isopropenyl-5-methylhex-4-enal
73	10.5997	0.248	Benzaldehyde, 3‑methoxy-
75	10.7771	1.1155	Urea, triethylnitroso-
76	10.8973	0.2125	Pyrazine, 2,5‑diethyl-
77	11.1376	0.2478	N,N'-Trimethyleneurea
78	11.2062	0.8315	3-Morpholinopropyl 2,4-dihydroxybenzoate
79	11.6182	0.3556	1-.beta.-d-Ribofuranosyl-3-[5-*tetraazolyl*]−1,2,4-triazole
80	11.6869	0.8876	Tetraacetyl-d-xylonic nitrile
81	11.7956	0.2718	2-Formylbenzeneboronic acid
82	11.8643	0.2533	Pyrazine, 3,5-dimethyl-2-propyl-
83	12.1046	0.2266	2,2′-Bis(4,5-dimethylimidazole)
84	12.2362	0.2891	Pyrimidine-2,4‑dione, hexahydro-3,6-dimethyl-1-(4-morpholinobutyl)-
86	12.7054	0.2138	2.alpha., 4a.beta., 8a.beta.-Decahydro-2-naphthalenol
88	13.9928	0.2862	Azulene
89	14.1016	0.2618	3-Undecene, 9-methyl-, (E)-
91	14.6337	0.2426	2-Bornanone oxime
92	15.1029	0.6078	2,5-Cyclohexadien-1-one, 3,5-dihydroxy-4,4-dimethyl-
93	15.2288	0.2668	2,5-Furandione, dihydro-3-(2-methyl-2-propenyl)-
94	15.9784	0.3259	Diethyldithiophosphinic acid
95	16.0184	1.1819	5,10-Diethoxy-2,3,7,8-tetrahydro-1H,6H-dipyrrolo[1,2-*a*:1′,2′-*d*]pyrazine
96	21.666	0.3139	Tetrasiloxane, decamethyl-

**Table 12:** GC–MS results of bio-oil from plantain peel with heterogeneous catalysis (HTC) at 450–550 °C.

Peak	Retention Time	Area Percentage	IUPAC Nomenclature
1	3.1211	2.1394	Pyrimidine, 5-methyl-
2	3.3099	1.6877	Cyclopentanone, 2-methyl-
3	3.3671	1.7091	2-Octanol, 2,6-dimethyl-
4	3.4244	3.2502	Butanoic acid
5–10	3.5159–38,306	9.5123	2-Furanmethanol
11	3.9508–4.0309	0.1714	1,2,3,4-Pentadecanetetrol, [2R-(2R*,3S*,4S*)]-
12–13	4.0309–4.0709	6.121	2-Cyclopenten-1-one, 2-methyl-
14	4.1739	1.1695	Hexanoic acid
15	4.3571	0.4348	Pyridine, 2,4-dimethyl-
16	4.4486	1.5871	Butanoic acid, 4‑hydroxy-
17	4.5001	0.4248	2-Cyclopenten-1-one, 2,3-dimethyl-
18	4.6546	0.4109	2-Pyridinecarboxylic acid, 6-methyl-
19–20	4.7748–4.8377	2.9781	2-Cyclopenten-1-one, 3-methyl-
21	4.975	5.0922	2,4-Dimethylfuran
22	5.141	5.0711	Phenol
23	5.2611	0.4308	1,2-Benzenediamine, 4-methyl-
24	5.4385	0.8541	Butyl aldoxime, 3-methyl-, anti
25	5.5758	2.799	Pyrimidine, 2-methyl-
26	5.7532	5.1888	2-Cyclopenten-1-one, 2,3-dimethyl-
27	5.9134	1.2352	Phenol, 2-methyl-
28	5.9649	0.1993	Imidosulfurous difluoride, methyl-
29	6.0393	1.4005	Phenol, 2-methyl-
30	6.1537	0.4089	2-Amino-4-methylpyrimidine
31–32	6.1938–6.2911	2.3231	p-Cresol
33	6.3483	0.9618	Phenol, 3-methyl-
34	6.4169	2.5688	2H-Pyran, tetrahydro-2-(12-pentadecynyloxy)-
35	6.4913	0.4508	4-Aminocyclohexanone, N-acetyl-
36	6.5428	0.2373	1H-Pyrazole, 1,3,5-trimethyl-
38	6.6516	0.3382	1,3-Benzenediamine
39	6.7546	1.4717	3-Piperidinone, 1,6-dimethyl-
40	6.8919	2.278	1,1-Dimethyl-1-silacyclobutane
41	7.012	1.1604	2,4-Heptadiene, 2,4-dimethyl-
42	7.1494	2.0393	1-Methyl-2-aminomethylimidazole
43	7.2581	1.3277	Phenol, 2,5-dimethyl-
44	7.4069	0.202	1-Pyrrolidinecarbonitrile
45	7.4812	0.7753	Phenol, 4-ethyl-
46–47	7.6071–7.6415	0.7741	Hexahydroindole
48	7.6872	0.4798	Divinylbis(cyclopropyl)silane
49	7.7216	0.2835	1,3-Cyclopentanedione, 4‑hydroxy-2-pentyl-
51	7.8303	0.9863	Phenol, 3,5-dimethyl-
52	7.8818	0.946	Cyclohexanol, 3,3,5-trimethyl-
53	7.9161	0.7073	3-Methylthiophene-2-carbonitrile
54	8.0935	2.0078	1,4:3,6-Dianhydro-.alpha.-d-glucopyranose
55	8.185	1.0709	Phenol, 2-ethoxy-
56	8.2938	1.5641	3,4,5-Trimethylpyrazole
57	8.3338	1.279	1H-Pyrazole, 1,3,5-trimethyl-
58	8.3968	0.5317	3,4,5-Trimethylpyrazole
59	8.4425	0.7855	1-Methoxy-1,3-cyclohexadiene
60	8.5799	0.8353	4,6-Dimethyl-2-pyrimidone
63	8.8202	0.148	Phenol, 2,3,5-trimethyl-
64	8.8946	0.6677	5-Fluoro-m-xylene
65	8.969	0.5068	2,4-Bis(hydroxylamino)pyrimidine
66	9.0662	0.7437	2,6-Dimethylfluorobenzene
67	9.1349	1.7257	Indole
68	9.3695	0.29	Benzocycloheptatriene
69	9.4954	0.1804	1H-Imidazole-4-ethanamine, 1,5-dimethyl-
70	9.6155	0.1905	2-Furancarboxaldehyde, 5-methyl-
71	9.7185	0.2786	Benzaldehyde, 4-ethoxy-
72	9.7872	0.2776	Phenol, 2,6-dimethoxy-
73	9.9016	1.2869	trans-4a-Methyl-decahydronaphthalene
74	10.0332	0.1705	3-Pyridinol, 2,6-dimethyl-
75	10.1076	0.1598	Pyrazine, 2-(n-propyl)-
76	10.182	0.5943	2-Tetradecene, (E)-
77	10.2678	0.4387	1H-Indole, 2-methyl-
78	10.4796	0.1828	7-Methyl-trans-8-thiabicyclo[4.3.0]nonane
79	10.5883	0.2385	Pyrazine, methoxy‑
80	10.6913	0.4516	2,4,6-Trimethylbenzonitrile, N-oxide
81	10.7714	0.4964	2,4-Imidazolidinedione, 5-(4-hydroxybutyl)-
82	11.1776	0.4487	Mexiletine acetate
83	11.3379	0.6202	1-Tridecene
84	11.4237	0.6241	Pentadecane
85	11.4752	0.2358	1-Pentadecene
86	11.6125	0.3928	16-Hydroxyhexadecanoic acid
87	11.7842	0.2236	2-Formylbenzeneboronic acid
88	11.87	0.1661	Phenol, 2,4-dimethyl-
89	11.9329	0.1489	Hydrazinecarboxamide, 2-(2,6-cyclooctadien-1-ylidene)-
90	12.5223	0.1535	Benzaldehyde, 2,4-dimethoxy-
91	13.1117	0.1625	4-Morpholinepropanoic acid, methyl ester
92	13.4721	0.1668	Trichloroacetic acid, undecyl ester
93	13.8555	0.3379	2-Dodecene, (Z)-
94	14.0901	0.1845	2-Exo‑hydroxy-5-ketobornane
95	14.9713	0.2001	2,6R-Diethyl-3,5S-dimethyl-3,4-dihydro-2H-pyran
96	15.0972	0.4251	2-Ethyl-1,3,4-trimethyl-3-pyrazolin-5-one
98	15.9727	0.2311	Formic acid, (2-fluoro-5-nitrophenyl)methyl ester
99	16.0241	1.4771	n-Hexadecanoic acid
100	21.6717	0.1891	1,2-Benzisothiazol-3-amine tbdms

**Table 13:** GC–MS results of bio-oil from plantain peel with heterogeneous catalysis (HTC) at 550–650 °C.

Peak	Retention Time	Area Percentage	IUPAC Nomenclature
1	3.3042	0.6183	Cyclohexanone
2	3.4644	0.7971	2-Furanmethanol
3	3.5445	0.9279	Pyridine, 3-methyl-
4	3.6132	1.3858	Benzene, 1,3-dimethyl-
5	3.8077	0.3183	Pyridine, 2,4-dimethyl-
6	3.8592	1.0083	1,3,5,7-Cyclooctatetraene
7	4.0366	0.8787	2-Cyclopenten-1-one, 2-methyl-
8	4.3341	0.5615	Pyridine, 2,4-dimethyl-
9	4.42	0.3555	Cyclopentanone, 2-ethyl-
10	4.5115	0.3678	Pyridine, 2,3-dimethyl-
11	4.7175	0.7871	Benzene, 1-ethyl-3-methyl-
12	4.7862	0.7801	2-Cyclopenten-1-one, 3-methyl-
13	4.9578	3.836	Phenol
14	5.038	0.5918	Cyclooctane, methyl-
15	5.1467	2.2559	Benzene, 2-propenyl-
16	5.1924	1.2052	Benzene, 1-ethenyl-4-methyl-
17	5.53	0.953	Benzene, 1-ethyl-3-methyl-
18	5.5987	1.634	D-Limonene
19	5.7589	0.8992	2-Cyclopenten-1-one, 2,3-dimethyl-
20	5.8447	1.6896	Indene
21	5.9134	3.465	Phenol, 2-methyl-
23	6.2052	4.3095	p-Cresol
24	6.3654	0.3822	1-Nonene
25	6.4112	0.5368	Benzene, (2-methyl-1-propenyl)-
26	6.4799	0.6809	Undecane
27	6.6344	1.2455	Benzenemethanol, 4-methyl-
28	6.8404	0.4371	Benzene, 1,2,4,5-tetramethyl-
29	7.0292	1.3299	Phenol, 2-ethyl-
30	7.1894	2.9647	Phenol, 2,3-dimethyl-
31	7.2524	2.1109	1H-Indene, 1-methyl-
33	7.4583	3.8448	Phenol, 2-ethyl-
34	7.5842	1.1404	Phenol, 2,3-dimethyl-
35	7.6929	0.9669	1-Decene
36	7.7444	2.264	Naphthalene
39	8.1393	0.7439	Phenol, 2-propyl-
40–41	8.21948.2651	1.1911	Phenol, 2-ethyl-5-methyl-
44	8.4883	0.4793	2-Ethyl-1-H-indene
45	8.6485	2.1757	Phenol, 3,4,5-trimethyl-
46, 48	8.7458, 8.8946	1.7955	Phenol, 2,4,6-trimethyl-
47	8.8087	1.0017	Phenol, 2,3,5-trimethyl-
49	8.9747	0.9934	1-Tridecene
50	9.0777	0.9808	Tridecane
51	9.1349	3.7593	Indole
52	9.2951	0.7095	2,5-Diethylphenol
53	9.3809	1.3401	Naphthalene, 1-methyl-
54	9.7185	0.6829	Phenol, 3,5‑diethyl-
55	9.9016	0.7306	2-Acetyl-3-ethylpyrazine
56	10.0103	0.3726	2-Methoxy-4‑chloro-phenol
57	10.1133	0.5203	1,2,3-Trimethylindene
58	10.1992	0.9818	2-Tetradecene, (E)-
61	10.4166	0.3707	Benzenemethanol, 4‑chloro-.alpha.-methyl-
62	10.4967	0.9566	Naphthalene, 2,6-dimethyl-
63	10.5883	0.4585	Benzaldehyde, 4-(1-methylethyl)-
65–66	10.7199–10.9201	0.8922	Naphthalene, 2,6-dimethyl-
67	11.0517	0.551	Biphenylene
68	11.1032	0.7689	Naphthalene, 2,7-dimethyl-
69	11.3607	2.0746	1-Pentadecene
70	11.4466	1.8215	Pentadecane
71	11.4923	0.6108	1-Tridecene
72	11.6182	0.4249	1-Pentadecene
73	12.1504	0.429	Benzene, 1-(1-methylethenyl)−3-(1-methylethyl)-
74	12.282	0.3892	1H-Benzimidazole, 2-(1-methylethyl)-
75	12.3392	0.3455	Fluorene-9-methanol
76	12.5223	0.6088	Hexadecane
77	12.7683	0.3412	1H-Phenalene
78	12.9343	0.3619	Fluorene
79	13.4893	0.4417	1-Heptadecene
80	13.558	0.6425	Heptadecane
81–82	13.7296–13.8326	1.0709	9H-Fluorene, 2-methyl-
83	13.8727	0.3736	5-Tetradecene, (E)-
84	14.1359	0.4161	Stilbene
85	14.1702	0.512	9H-Fluoren-9-one
86	14.5879	0.6086	Phenanthrene
87	15.5206	0.618	Hexadecanenitrile
88	15.7209	0.5961	Hexadecanoic acid, methyl ester
89	15.9097	0.3547	5H-Dibenzo[*a,d*]cyclohepten-5-ol, 10,11-dihydro-
90	16.1214	3.9789	n-Hexadecanoic acid
92	17.5462	0.4209	9,12-Octadecadienoic acid (Z,Z)-
93	17.592	0.6042	9-Octadecenoic acid, (E)-
94	17.6263	0.5066	Octadec-9-enoic acid
95	17.7636	0.3422	Octadecanoic acid
96	17.941	0.3316	Tetradecanamide
97	24.4469	0.4385	Carbonic acid, monoamide, N-methyl-N-phenyl-, butyl ester
98	24.5384	0.3839	Ginsenol

**Table 14:** GC–MS results of bio-oil obtained from plantain peel with heterogeneous catalysis (HMC) at 300–400 °C.

Peak		Retention Time	Area Percentage	IUPAC Nomenclature
2	3.2985		1.0794	Cyclopentanone, 2-methyl-
3	3.3672		1.0098	(*R*)-(+)−3-Methylcyclopentanone
4–8	3.4244–3.865		6.8298	2-Furanmethanol
9	3.9565		0.2046	Hexanoic acid, 2-methyl-
10–11	4.0366–4.0767		8.93	2-Cyclopenten-1-one, 2-methyl-
12	4.2083		0.9199	Hexanoic acid
13	4.2713		0.2566	2-Butenoic acid, 3-methyl-
14	4.3628		0.2907	Pyridine, 3,5-dimethyl-
15	4.4143		0.8227	Cyclohexene, 1,2-dimethyl-
16	4.4887		2.3826	Butyrolactone
17	4.6603		0.3929	2-Pyridinecarboxylic acid, 6-methyl-
18–19	4.8034–4.9064		5.7788	2-Cyclopenten-1-one, 3-methyl-
20	4.9464		2.2616	2,4-Dimethylfuran
21	5.0037		3.2426	Formic acid phenyl ester
22	5.181		5.8763	Phenol
23	5.2898		0.986	Hexanoic acid
24	5.4042		0.2072	Heptane, 4-propyl-
25	5.4614		0.7888	Allyldiethylamine
26	5.6216		0.7397	Bicyclo[3.3.1]nonan-2-one
27	5.7876		7.6945	1,2-Cyclopentanedione, 3-methyl-
28	5.8391		0.2683	Pyrazine, 1,4-dioxide
29	5.8734		0.277	4(1H)-Pyrimidinone, 6‑hydroxy-
30	5.9192		0.9537	Phenol, 2-methyl-
31	5.9936		0.2596	2-Propenal, 3-(dimethylamino)-
32	6.0565		0.8546	Phenol, 2-methyl-
34–35	6.1938–6.3083		3.5729	p-Cresol
36	6.4284		3.0881	Phenol, 2‑methoxy-
37	6.5085		0.2998	Piperidine, 3,5-dimethyl-
38	6.6516		3.2441	2-Hexen-1-ol, (E)-
39	6.7946		1.1924	2,2-Dimethyl-1-oxa-spiro[2.4]heptane
40	6.8576		0.2427	Maltol
41	6.9377		2.1252	2-Cyclopenten-1-one, 3-ethyl-2‑hydroxy-
42	7.0121		1.1625	1,3-Hexadiene, 3-ethyl-2-methyl-, (Z)-
43	7.1723		1.4207	3-Buten-2-one, 4-(1-aziridinyl)-
44	7.2753		1.033	Benzene, ethoxy-
45	7.3554		0.7182	Pyrazine, 3,5‑diethyl-2-methyl-
46–48	7.4298–7.5557		1.4242	3-Dimethylaminoacrylonitrile
49	7.733		0.5727	3-Fluoropropiophenone
50	7.8418		0.4061	Bicyclo[3.3.1]nonan-2-ol
51	7.899		0.8708	Valeric acid, tridec‑2-ynyl ester
52	7.9505		1.1972	3-Methylthiophene-2-carbonitrile
53	8.1164		3.0098	1,4:3,6-Dianhydro-.alpha.-d-glucopyranose
54	8.1908		0.3902	1H-Pyrazole, 1,3,5-trimethyl-
55	8.351		0.9638	Cyclohexene, 1-methyl-3-(formylmethyl)-
56	8.4597		0.7711	1H-Imidazole-4-ethanamine, .beta.,.beta.-dimethyl-
57	8.62		0.3395	1H-Pyrazole, 1,3,5-trimethyl-
58	8.6657		1.0614	2,5-Dimethylhex-5-en-3-yn-2-ol
59	8.7744		0.9721	1H-Imidazole, 2,4,5-trimethyl-
60	8.9175		0.6196	Benzene, (fluoromethyl)-
61	8.9804		0.264	2,6-Dimethylfluorobenzene
62	9.1063		0.6899	3-Fluoro-o-xylene
63	9.1407		0.7093	Benzyl nitrile
64	9.1864		0.6191	Pyrrolidine, 1-(1-butenyl)-
65	9.2837		0.238	Naphthalen-4a,8a-imine, octahydro-
66	9.3524		0.2696	3,4,5,6,7,8-Hexahydro-2H-chromene
67	9.7186		0.2321	4,6-Dimethyl-2-pyrimidone
68	9.793		0.4659	Phenol, 2,6-dimethoxy-
69	9.9017		1.0583	5-t-Butyl-hexa-3,5‑dien-2-one
70	9.9989		0.4966	2-Butenediamide, (E)-
71	10.0447		0.231	Benzenemethanol, 3-amino-
72	10.1935		0.64	1-Propanamine, N-cyclohexylidene-
74	11.2063		0.5996	2H-1,4-Oxazine-4-acetic acid, tetrahydro-
75	11.418		0.2728	8-Methyloctahydrocoumarin
76	11.6125		0.4395	Benzenamine, 3‑methoxy-
77	12.2534		0.3802	Mexiletine acetate
78	12.3163		0.256	Phenol, p-(2-methylallyl)-
81	14.0215		0.4257	2-Amino-4,5-dimethylthiazole
82	14.1188		0.3841	L-Proline, N-(hexanoyl)-, decyl ester
84	14.8683		0.247	2-Hydroxy-3,5,5-trimethyl-cyclohex-2-enone
85	14.937		0.1931	Cyclopentanecarboxylic acid, 4-methylene-2-phenyl-, methyl ester, trans-
86	15.1144		0.7091	Phenol, 3,5-dimethoxy-
87	15.8983		0.2761	Pyrrolo[1,2-*a*]pyrazine-1,4‑dione, hexahydro-3-(2-methylpropyl)-
88	16.0242		2.1486	5-Isopropylidene-3,3-dimethyl-dihydrofuran-2-one
89–90	17.5519–17.5863		0.5359	9-Octadecenoic acid, (E)-
91	20.4015		0.2105	2-Ethylacridine
92	21.666		0.2878	Cyclotrisiloxane, hexamethyl-

**Table 15:** GC–MS results of bio-oil from plantain peel with heterogeneous catalysis (HMC) at 400–500 °C.

Peak	Retention Time	Area Percentage	IUPAC Nomenclature
1	3.1269	0.4985	5-Vinyl-pyrazole
2	3.1784	1.4186	N,N-Dimethylaminoethanol
3	3.2985	0.6537	Cyclopentanone, 2-methyl-
4	3.3615	0.8483	.beta.-d-Glucopyranose, 1,6-anhydro-
5	3.4358	2.96	1,6:2,3-Dianhydro-4-O-acetyl-.beta.-d-mannopyranose
6–7	3.5732–3.8535	5.6362	2-Furanmethanol
8	4.0366	3.8195	2-Cyclopenten-1-one, 2-methyl-
9	4.2026	0.4344	Pyrazine, 2,6-dimethyl-
10	4.3628	0.245	Pyridine, 3,5-dimethyl-
11	4.4143	0.5864	2,4-Hexadiene, 2,3-dimethyl-
12	4.483	2.852	Butyrolactone
13	4.6546	0.2724	1,3-Dimethyl-pyridinium chloride
14–15	4.7862–4.9121	3.3514	2-Cyclopenten-1-one, 3-methyl-
16	4.9979	2.8429	Cyclopentane, ethylidene-
17	5.181	5.7758	Phenol
18	5.3012	1.5328	2,4,6-Cycloheptatrien-1-one, 2‑hydroxy-
19	5.4671	1.2251	Silane, triethyl-
20	5.5015	0.2912	Butanal, 3-methyl-, oxime
21	5.6216	1.0728	3-Aminopyridine
23,25	5.9135, 6.0565	1.5284	Phenol, 2-methyl-
24	5.9821	0.3458	2-Pyrrolidinone, 1-methyl-
27–28	6.1881–6.3083	3.8921	p-Cresol
29	6.4055	0.8744	Phenol, 2‑methoxy-
30	6.4971	0.7436	Tetrahydrofuran, 2-ethyl-5-methyl-
31	6.6516	3.995	2-Hexen-1-ol, (E)-
32	6.8003	1.889	3-Piperidinone, 1,6-dimethyl-
33	6.8633	0.3536	Maltol
35	7.0178	0.6003	1,3-Hexadiene, 3-ethyl-2-methyl-, (Z)-
37	7.281	1.0051	Borolo[1,2-*a*]borine, octahydro-
40	7.4927	0.823	3-Dimethylaminoacrylonitrile
41	7.6186	0.3067	1H-Imidazole, 2,4-dimethyl-
43	7.8418	0.5969	Phenol, 3,5-dimethyl-
44	7.9104	1.5875	10-Methylundecan-4-olide
45	7.9562	1.3832	5-Dimethylaminopyrimidine
46	8.1221	3.5738	1,4:3,6-Dianhydro-.alpha.-d-glucopyranose
47	8.1965	0.2562	1H-Pyrazole, 1,3,5-trimethyl-
48	8.3453	1.0452	Cyclohexanone, 2-(2-propenyl)-
49	8.4597	0.8755	1H-Pyrazole, 1,3,5-trimethyl-
50	8.5684	0.4032	Furan, 2,3,5-trimethyl-
51	8.6714	0.4594	4-Pyrazolylmethanamine, 1-ethyl-
52	8.7802	0.5452	3-(2-Hydroxy-2-methyl-propyl)-cyclohex-2-enone
53	8.906	0.4404	Phenol, 3-amino-
54	8.9804	0.6348	2,6-Dimethylfluorobenzene
55	9.1063	0.4298	1,1-Dimethyl-4-methylenecyclohexane
56	9.1464	1.2177	Indole
57	9.1921	0.8695	Pyrrolidine, 1-(1-butenyl)-
58	9.2837	0.3335	Guanine
59	9.3924	0.4062	2-Cyclohexen-1-one dimethylketal
60	9.4954	0.2048	8-Hydroxyisotrichodermin
61	9.627	0.2769	Cyclopentanecarboxaldehyde, 2-methyl-3-methylene-
62	9.7128	0.3796	4,6-Dimethyl-2-pyrimidone
63	9.7929	0.4589	Phenol, 2,6-dimethoxy-
64	9.8673	0.3453	1-Methoxy-1,3-cyclohexadiene
65	9.9074	0.7033	5-t-Butyl-hexa-3,5‑dien-2-one
66	10.0047	0.7363	2,4-Imidazolidinedione, 5-methyl-
68	10.1134	0.2577	Benzene, 2-fluoro-1,3,5-trimethyl-
69	10.1935	0.7519	1-Propanamine, N-cyclohexylidene-
72	11.1491	0.4889	2,3-Dimethylperhydro-1,3-oxazine
73	11.212	1.4396	Ethiolate
74	11.3322	0.222	Adenine
75	11.418	0.3298	1,4-Benzenediol, 2-methyl-
77	11.6812	0.2299	Butanoic acid, 3‑hydroxy-
78	11.87	0.2709	Pyrazine, 2,5-dimethyl-3-propyl-
79	12.2591	0.3592	Morpholine, 4-(2-chloroethyl)-
80	12.3106	0.3092	1,2,3,4-Tetrahydro-pyridine-2,5-dicarbonitrile
82	12.7112	0.3814	N-(2,4,6-Trimethyl-3-pyridyl)acetamide
83	13.1517	0.2144	2,4—Nonanedione
84	13.4779	0.3365	N-Allyl-2-pyridone
85	13.8727	0.3123	3-Pyrrolidin-2-yl-propionic acid
86	14.01	0.4728	2-Cyclohexen-1-one, 4,4,5-trimethoxy-
93	17.5519	0.332	6-Octadecenoic acid
94	17.5863	0.2663	E-9-Tetradecenoic acid
95	20.4015	0.2213	Hexadecanoic acid, 2‑hydroxy-1-(hydroxymethyl)ethyl ester
96	21.6717	0.3286	1,2-Benzisothiazol-3-amine tbdms

**Table 16:** GC–MS results of bio-oil from plantain peel with heterogeneous catalysis (HMC) at 500–600 °C.

Peak	Retention Time	Area Percentage	IUPAC Nomenclature
2	3.1612	1.8028	3-Aminopyridine
3	3.2985	0.4133	Cyclopentanone, 2-methyl-
4	3.4187	5.0426	Pentanamide, 4-methyl-
5	3.5274	0.7266	Butanoic acid
6	3.5732	0.6633	Pyridine, 3-methyl-
7	3.8478	2.7835	2-Furanmethanol
8	3.9108	0.6219	Methyl .beta.-d-galactopyranoside
11	4.1911	0.4502	Pyrazine, 2,6-dimethyl-
12	4.4715	3.2967	Butyrolactone
13	4.6546	0.4525	1,3-Dimethyl-pyridinium chloride
14–15	4.7805–5.181	2.9826	2-Cyclopenten-1-one, 3-methyl-
16	5.181	4.5182	Phenol
17	5.2955	1.113	2-Pyridinamine, 4,6-dimethyl-
18	5.4671	1.2155	Oxirane, [(1-methylethoxy)methyl]-
19	5.6044	1.3844	2-Aminopyridine
20	5.7704	6.4035	2-Cyclopenten-1-one, 2,3-dimethyl-
21	5.9077	1.1857	Phenol, 2-methyl-
22	5.9707	0.6241	2-Pyrrolidinone, 1-methyl-
23	6.0565	0.8545	Phenol, 2-methyl-
24	6.0908	0.9183	1,2,4-Cyclopentanetrione, 3-methyl-
25	6.1824	1.1415	p-Cresol
26	6.3254	2.7442	Phenol, 3-methyl-
27	6.4284	3.1639	Mequinol
28	6.5028	1.2376	2-Pyrrolidinone
29	6.6916	4.4246	2-Hexen-1-ol, (E)-
30	6.8003	2.6877	Methylamine, N-(1-propylbutylidene)-
31	6.8576	0.7369	Maltol
32	6.932	2.7086	1H-Imidazole-4-carboxylic acid, methyl ester
34	7.1666	3.3617	4(1H)-Pyridinone, 2,3-dihydro-1-methyl-
35	7.281	0.695	Phenol, 2,5-dimethyl-
36	7.3497	0.3255	6-Amino-1-methylpurine
37	7.4412	0.5247	1H-Pyrazole, 1,5-dimethyl-
38	7.487	0.8656	2-Acetyl-1,4,5,6-tetrahydropyridine
39	7.5328	0.6965	3-Dimethylaminoacrylonitrile
40–41	7.5671–7.6129	1.2037	1H-Imidazole, 2,4-dimethyl-
42	7.7159	0.4683	1-Buta-1,3-dienyl-pyrrolidine
43	7.8475	0.69	Pyrazole, 1-vinyl-3,5-dimethyl-
44	7.9161	0.8918	10-Methylundecan-4-olide
45	8.1278	3.4431	1,4:3,6-Dianhydro-.alpha.-d-glucopyranose
50	8.6714	0.5251	Dicyclobutylidene oxide
51	8.7802	0.4524	Octanohydrazide, N2-(2-furfurylidene)-
52	8.9175	0.5341	Phenol, 3-amino-
53	8.9804	0.6045	2,6-Dimethylfluorobenzene
54	9.1063	0.189	Creosol
55	9.1521	0.7425	Benzene, (fluoromethyl)-
56	9.2894	0.3339	Guanine
57	9.3581	0.3384	Imidazole, 1,4,5-trimethyl-
58	9.4038	0.4697	4H—Cyclopenta[*b*]pyridin-4-one, octahydro-
59	9.4954	0.2334	8-Hydroxyisotrichodermin
60	9.627	0.2558	1-Azabicyclo[2.2.2]octane, 4-methyl-
62	9.7929	0.3507	Phenol, 2,6-dimethoxy-
63	9.8673	0.3182	2,3,4-Trimethylpyrrole
64	9.9131	0.3096	4-Hydroxy-2,4,5-trimethyl-2,5-cyclohexadien-1-one
65–66	10.0047–10.0504	0.5963	2,4-Imidazolidinedione, 5-methyl-
67	10.1935	0.6319	1-Propanamine, N-cyclohexylidene-
68	10.5997	0.197	2,5,10-Undecatrienoic acid, methyl ester
70	10.7943	0.309	1-(4-Acetoxyphenyl)−3-morpholino-propan-1-one
71	11.0747	0.2427	.+/-.−1,3,4,5,6,7-Hexahydro-2H-pyrindin-2-one
72	11.149	0.1822	1,4-Benzenediol, 2-methyl-
73	11.212	0.2378	Ethiolate
74	11.418	0.3149	1-Cyclohexene-1-acetaldehyde, 2,6,6-trimethyl-
75	11.7155	1.7351	Formamide, N-[1-[(1-*cyanopropyl*)*hydroxyamino*]butyl]-
76	11.8071	0.177	2-Formylbenzeneboronic acid
77	11.87	0.3843	Pyrazine, 3,5-dimethyl-2-propyl-
78	12.2648	0.2006	Indole-2(3H)-one, 1-(4-morppholylmethyl)−3spiro(4-chloromethyl-1,3-dioxalan-2-yl)-
79	12.4422	0.5975	2-Amino-3,4:5,6-bis(trimethylene)pyridine
80	12.7111	0.2042	2,3,5-Trimethyl-6-butylpyrazine
81	13.4836	0.1857	Tetrahydrofuran, 2-isobutenyl-4-vinyl-
82	13.8784	0.3215	2-Tridecenal, (E)-
83	14.0215	0.4042	2-Amino-4,5-dimethylthiazole
84	14.1302	0.3833	Nonanoic acid, 3-methylbutyl ester
85	14.3247	0.3235	Pyrrolo[1,2-*a*]pyrazine-1,4‑dione, hexahydro-
86	15.1201	0.8736	Phenol, 3,5-dimethoxy-
90	17.5519	0.2644	Octadec-9-enoic acid
91	17.5863	0.2375	E-11-Hexadecenal
92	20.3958	0.1898	1,2-Bis(trimethylsilyl)benzene

**Table 17:** GC–MS results of bio-oil from plantain peels with heterogeneous catalysis (HMC) at 600–700 °C.

Peak	Retention Time	Area Percentage	IUPAC Nomenclature
2	3.184	2.1993	Methyl 3-dimethylaminopropionate
3	3.3099	0.3396	3-Pentanol, 2-methyl-
4	3.3729	0.5843	Octanoic acid
5	3.4301	2.0364	2-Hydroxy-2,6-dimethyl‑hept-6-en-3-one
6–7	3.7334–3.8249	2.0197	2-Furanmethanol
8	3.968	1.397	Propanal, 3‑methoxy-
9	4.0366	1.3281	2-Cyclopenten-1-one, 2-methyl-
10	4.0881	1.7262	1,4-Pentadiene, 2,3,3-trimethyl-
11	4.1167	2.7086	1,3-Benzenediamine
12	4.3399	1.5071	Pentanoic acid
13	4.3857	0.4549	2-Butenoic acid, 3-methyl-
14	4.4658	2.4577	Butanoic acid, 4‑hydroxy-
15	4.5459	0.347	2-Propanamine, N,N-dimethyl-
16	4.6546	0.2515	Ketone, methyl 6-methyl-2-pyridyl
17–18	4.7976–4.8491	9.6941	2-Cyclopenten-1-one, 3-methyl-
19–20	4.9979–5.1867	7.5727	Phenol
21	5.3527	0.9526	Hexanoic acid
22	5.5129	1.2681	Silane, trimethyl-
23	5.5472	0.6057	Oxirane, [(1-methylethoxy)methyl]-
24	5.6159	1.3411	Pyrazine, methyl-
25	5.7532	3.0115	2-Cyclopenten-1-one, 2,3-dimethyl-
26,28,30	5.9077, 6.0622, 6.3254	2.8665	Phenol, 2-methyl-
27	5.9764	0.4228	2-Pyrrolidinone, 1-methyl-
29	6.1995	1.5374	2-Amino-4-methylpyrimidine
31	6.4284	1.9793	Phenol, 2‑methoxy-
33	6.6058	1.4107	2-Pyrrolidinone
34	6.7145	1.825	Hexanal, 2-ethyl-
35	6.869	2.6186	Benzene-D6
36	6.9777	0.3624	Pentanamide, 4-methyl-
37	7.1665	3.3848	3-Pyridinol-1-oxide
38	7.2581	3.6988	Butanoic acid, tridec‑2-ynyl ester
39	7.487	1.2	3-Buten-2-one, 4-(1-aziridinyl)-
40–41	7.5671–7.6357	1.2124	3-Dimethylaminoacrylonitrile
42	7.6872	0.3743	1H-Imidazole, 2,4-dimethyl-
43	7.7445	0.2878	Pyrazole, 1,4-dimethyl-
44	7.8703	0.2969	1H-Imidazole, 4,5-dimethyl-
45	7.9218	0.9049	Cyclopropane, 1,1-dimethyl-2-(2-propenyl)-
47	8.0363	2.4455	7-[2-T*ertahydropyranyloxy*]−1-heptyne
48	8.1793	5.5131	1,4:3,6-Dianhydro-.alpha.-d-glucopyranose
49	8.351	0.9618	Oxirane, hexadecyl-
50	8.4254	1.0242	3-Methoxy-2,5-dimethylpyrazine
51	8.597	0.2551	Cyclohexanol, 2,3-dimethyl-
54	8.7916	0.5656	4(1H)-Quinolinone, octahydro-4a,8a-dimethyl-
55	8.8602	0.5872	1-Buta-1,3-dienyl-pyrrolidine
56	8.9518	0.9757	Verbenol
57	9.0605	0.4129	Phenol, 4-amino-
58	9.1006	0.3534	2,4-Bis(hydroxylamino)pyrimidine
59	9.1635	0.4215	Benzonitrile, 4-methyl-
60	9.2322	0.9137	Pyrrolidine, 1-(1-butenyl)-
61	9.3924	0.6416	Imidazole, 1,4,5-trimethyl-
62	9.524	0.4431	Phenol, 4-amino-3-methyl-
63	9.6728	0.2394	Cyclodecylamine
64	9.7586	0.6177	Benzenamine, 3‑methoxy-
65	9.8902	0.2448	1-Methoxy-1,3-cyclohexadiene
66	10.0847	0.3098	Imidazole, 1,4,5-trimethyl-
67	10.2106	1.5464	1-Methoxy-1,4-cyclohexadiene
69	10.8515	0.8776	4-(2-((4-Fluorophenyl)sulfonyl)ethyl)morpholine
70	10.9144	0.4932	5-Ethylhydantoin
72	11.7784	0.6957	Octadecane, 1‑bromo-
73	11.8299	1.2032	1-Methylcycloheptanol
74	12.3335	0.2968	S-[1-Phenyl-2-[2,2-*dimethylpropyl*]aminoethyl] thiosulfate
75	12.6139	0.223	9-Borabicyclo[3.3.1]nonane, 9-ethyl-
76	12.8141	0.2591	7-Oxabicyclo[4.1.0]heptane, 2-methylene-
77	13.907	0.4278	1,2-Dimethyl-5-(2-cyanoethyl)piperid-4-one
78	14.0329	0.4913	1H-Azepine, hexahydro-1-nitroso-
79	14.1588	0.5653	2-Tridecenal, (E)-
85	17.5519	0.2717	E-9-Tetradecenoic acid
86	20.4015	0.1959	Hexadecanoic acid, 2‑hydroxy-1-(hydroxymethyl)ethyl ester
87	21.6717	0.3088	1,2-Benzisothiazol-3-amine tbdms

**Table 18:** Gas Chromatography-Mass Spectroscopy results of bio-oil from yam peel without catalyst (AOC) at 350–450 °C.

Peak	Retention Time	Area Percentage	IUPAC Nomenclature
1	3.2642	2.41	2-Cyclopenten-1-one
2	3.2985	0.783	2-Cyclopenten-1-one
3	3.453	4.2043	2-Furanmethanol
4	3.5846	0.6372	2-Furanmethanol
5	3.6018	0.5658	2-Furanmethanol
6	3.802	0.3712	2-Furanmethanol
7	3.8879	6.7902	2-Furanmethanol
8	4.0423	2.0214	2-Cyclopenten-1-one, 2-methyl-
9	4.0824	5.1398	4,4-Dimethyl-2-cyclopenten-1-one
10	4.2884	0.4165	Pentanoic acid
11	4.38	0.4215	Pyridine, 3,4-dimethyl-
12	4.42	0.1826	Pyridine, 2,4-dimethyl-
13	4.5688	5.7709	2-Propanone, methylhydrazone
14	4.7404	0.4148	Diethylcyanamide
15	4.7919	1.7935	2-Cyclopenten-1-one, 3-methyl-
16	4.8949	0.6557	2-Cyclopenten-1-one, 3-methyl-
17	4.9579	2.1158	3-Methylpyridazine
18	5.038	4.3539	Phenol
19	5.2211	7.1177	Phenol
20	5.4271	1.4745	2,4-Dimethyl-2-oxazoline-4-methanol
21	5.5358	6.507	1-Methyl-2-piperidinemethanol
22	5.6044	0.3016	Pyrazole, 1,4-dimethyl-
23	5.8448	7.1557	1,2-Cyclopentanedione, 3-methyl-
24	5.9249	0.6266	Phenol, 2-methyl-
25	6.0737	1.2262	Phenol, 2-methyl-
27	6.1938	1.0536	Phenol, 3-methyl-
28	6.3197	2.1017	p-Cresol
29	6.4227	2.8023	Phenol, 2‑methoxy-
30	6.5257	0.8798	Phenol, 2‑methoxy-
31	6.623	0.2226	2,4-Diaminophenol
32	6.726	0.4969	4-Amino-2,6-dihydroxypyrimidine
33	6.869	0.3295	3-Furancarboxylic acid, methyl ester
34	6.9491	2.212	2-Cyclopenten-1-one, 3-ethyl-2‑hydroxy-
35	7.0121	0.3396	2(1H)-Pyridinone, 3-methyl-
36	7.1666	0.378	1-Hexadecen-3-ol, 3,5,11,15-tetramethyl-
37	7.2295	0.2784	Pyrazole-3-carboxylic acid, 1-ethyl-
38	7.2696	0.777	Pyridine, 2,5-dimethyl-
39	7.4069	0.3056	Phenol, 3-ethyl-
40	7.4927	1.2078	Phenol, 4-ethyl-
41	7.6243	0.4815	3-Pyridinol, 2,6-dimethyl-
42	7.7731	0.1807	Creosol
43	7.8418	0.4814	3-Methoxy-5-methylphenol
44	7.9161	1.2343	2(3H)-Furanone, dihydro-5-pentyl-
45	8.0649	0.823	Catechol
46	8.1221	3.1126	1,4:3,6-Dianhydro-.alpha.-d-glucopyranose
47	8.1908	0.6985	1H-Pyrazole, 1,3,5-trimethyl-
48	8.2938	4.8508	[1,1′-Bicyclopentyl]−2-one
49	8.4025	0.1858	Phenol, 3,4,5-trimethyl-
50	8.8087	0.3524	1,4-Benzenediol, 2-methyl-
51	8.9175	0.6337	Ethanone, 1-(2,5-dihydroxyphenyl)-
52	9.0834	0.4856	Hydroquinone
53	9.3123	0.2198	1-(2-Propenyl)piperidine
55	9.7929	0.6832	Phenol, 2,6-dimethoxy-
56	9.9989	0.1901	m-Guaiacol
57	11.8929	0.2424	1-(4-methylthiophenyl)−2-propanone
58	12.2248	0.1876	Bicyclo[10.1.0]trideca-4,8-diene-13-carboxamide, N-(3-chlorophenyl)-
59	13.1918	0.2831	Triethyl citrate
60	16.0127	1.2299	n-Hexadecanoic acid
61	17.5004	0.3004	9,12-Octadecadienoic acid (Z,Z)-
62	17.5348	0.5523	9-Octadecenoic acid, (E)-
63	17.5691	0.4927	9-Octadecenoic acid, (E)-
64	17.7064	0.4207	Octadecanoic acid
66	24.4641	0.2571	1,2-Bis(trimethylsilyl)benzene
67	24.5613	0.2271	Trimethyl(4‑tert-butylphenoxy)silane
68	24.8131	0.2187	Cyclotrisiloxane, hexamethyl-
70	25.0477	0.3287	4-Methyl-2-trimethylsilyloxy-acetophenone

Fig. 1 provides a pictorial representation of the bio-oil yields obtained at each temperature ranges under the various processing conditions (absence of catalysts (AOC), HTC, and HMC).

(1) $$Bio-oil\left(wt\%\right)=\frac{weight\;of\;bio-oil}{weight\;of\;feed}\times \;100$$

(2) $$Biochar\;\left(wt\%\right)=\frac{weight\;of\;char}{weight\;of\;feed}\times \;100$$

(3) Non−condensables(wt%)=100−(bio−oil(wt%)+biochar(wt%))

## Experimental design, materials, and methods

### Pyrolysis of plantain and yam peels to obtain bio-oil

The pyrolysis reactor was purged and kept air-tight to prevent the interference of air/oxygen with the pyrolysis reaction [6]. 150 g of each of the sample feed (dried and pulverized plantain and yam peels) was weighed and fed into the reactor. The reactor was then switched on while the required input temperature (at a 100 °C interval.) was then appropriately set. As the temperature in the reactor gradually increased, vapors were steadily released and made to pass through the condensers for condensation into bio-oil. The temperature ranges for the collection of bio-oil were; 250–350 350–450, 450–550, and 550–650 °C for the pyrolysis plantain and yam peels without catalyst and using heterogeneous (HTC) catalysis, while 200–300 300–400, 400–500, 500–600, and 600–700 °C temperature ranges were used for the heterogeneous (HMC) catalysis. The collected liquid product (bio-oil), as well as the biochar, for each of the experiments, was weighed and recorded.

**Table 19:** Gas Chromatography-Mass Spectroscopy results of bio-oil from yam peels without catalyst (AOC) at 450–550 °C.

Peak	Retention Time	Area Percentage	IUPAC Nomenclature
1	3.1328	1.506	3-Aminopyridine
2	3.2529	1.0419	2-Cyclopenten-1-one
4	3.4532	5.7413	2-Furanmethanol
5	3.8194	0.4223	2-Furanmethanol
6	3.8823	1.2044	2-Furanmethanol
7	3.9224	3.4722	2-Furanmethanol
8	4.0426	2.0531	2-Cyclopenten-1-one, 2-methyl-
10	4.2428	0.5811	.beta.-l-Arabinopyranoside, methyl
11	4.4202	0.2908	1-Methylcycloheptene
12	4.5633	4.9448	Butanoic acid, 4‑hydroxy-
13	4.7979	1.0435	2-Cyclopenten-1-one, 3-methyl-
14	4.981	2.9082	Phenol
15	5.2098	7.676	Phenol
16	5.3128	0.4553	Heptanoic acid
17	5.5188	4.4732	2,4-Dimethyl-2-oxazoline-4-methanol
18	5.5989	0.332	Pyrazole, 1,4-dimethyl-
19	5.6676	0.2312	2-Aminopyridine
20	5.8335	6.8397	1,2-Cyclopentanedione, 3-methyl-
21	5.9022	0.7755	Phenol, 2-methyl-
22	6.0739	0.5826	Phenol, 2-methyl-
24	6.1997	0.9633	p-Cresol
25	6.3256	1.6465	Phenol, 3-methyl-
26	6.4229	3.3621	Phenol, 2‑methoxy-
27	6.5316	0.7184	2,5-Pyrrolidinedione, 1-methyl-
28	6.566	0.3488	Imidazole, 1,4,5-trimethyl-
29	6.6403	0.2458	(2,2-Dimethylcyclobutyl)methylamine
30	6.7319	0.5301	2H-Azepin-2-one, hexahydro-1-methyl-
31	6.7777	0.2431	Cyclohexane, 1,4-diethoxy-, trans-
32	6.8692	0.5772	Maltol
33	6.9493	1.7379	2-Cyclopenten-1-one, 3-ethyl-2‑hydroxy-
34	7.018	0.8809	2(1H)-Pyridinone, 3-methyl-
35	7.1782	0.8852	2,5-Dimethylcyclohexanol
36	7.2812	0.4616	Sulfuric acid, dimethyl ester
37	7.3613	0.2779	2-Thiophenecarboxylic acid, cyclobutyl ester
38	7.4986	1.0272	Phenol, 4-ethyl-
39	7.6302	0.4229	Hexahydroindole
40	7.7676	0.2305	1H-Pyrazole, 1,3,5-trimethyl-
41	7.8534	0.494	Phenol, o-amino-
42	7.9049	2.3233	4-Methylpentyl pentanoate
43	8.1281	8.6191	1,4:3,6-Dianhydro-.alpha.-d-glucopyranose
44	8.2768	2.5132	2-Cyclohexylpiperidine
45	8.3455	0.6721	3H-Indazol-3-one, 1,2,4,5,6,7-hexahydro-
46	8.4485	0.2701	1H-Pyrazole, 1,3,5-trimethyl-
47	8.5915	0.3631	1H-Imidazole, 1,2,4,5-tetramethyl-
48	8.8032	0.3129	5-Ethyl-2-furaldehyde
49	8.912	0.5006	1,4-Benzenediol, 2-methyl-
50	9.1466	1.7196	Hydroquinone
51	9.5585	0.2771	1-Octyl trifluoroacetate
52	9.6387	0.328	Tetrahydroionyl acetate
53	9.7931	0.5411	Phenol, 2,6-dimethoxy-
54	9.8733	0.5174	1,3-Benzenediol, 2-methyl-
55	10.331	0.2736	3-Pyridinol, 2,6-dimethyl-
56	10.4969	0.2337	1-Cyclohexene-1-carboxaldehyde, 2,6,6-trimethyl-
57	10.6057	0.2622	Benzaldehyde, 4‑methoxy-
58	11.3095	0.4578	3-Buten-2-one, 4-(2‑hydroxy-2,6,6-trimethylcyclohexyl)-
59	11.6413	0.5157	.beta.-d-Glucopyranose, 1,6-anhydro-
60	11.8702	0.4997	Pyrazine, 5‑butyl‑2,3-dimethyl-
61	12.019	0.2457	2-Butanamine, N-(2-furanylmethylene)-
62	12.3909	0.2678	1-(5,6-Dimethyl-2-pyrazinyl)propanone
63	13.8271	0.232	Benzenemethanol, 3-fluoro-.alpha.-methyl-
64	15.0745	0.2627	Phenol, 3,5-dimethoxy-
65	15.8642	0.2263	2-Hydroxy-3,5,5-trimethyl-cyclohex-2-enone
66	16.0015	1.7641	5,10-Diethoxy-2,3,7,8-tetrahydro-1H,6H-dipyrrolo[1,2-*a*:1′,2′-*d*]pyrazine
67	17.535	0.5301	9-Octadecenoic acid, (E)-
68	17.5693	0.4313	Octadec-9-enoic acid
69	17.7066	0.3401	Octadecanoic acid
74	24.9621	0.577	Tetrasiloxane, decamethyl-
75	25.0479	0.4006	Cyclotrisiloxane, hexamethyl-

**Table 20:** Gas Chromatography-Mass Spectroscopy results of bio-oil from yam peel without catalyst (AOC) at 550–650 °C.

Peak	Retention Time	Area Percentage	IUPAC Nomenclature
1	3.1671	1.1666	Fampridine
2	3.2987	0.3619	Cyclopentanone, 2-methyl-
3	3.436	3.4716	2-Furanmethanol
4	3.5448	0.4904	2-Furanmethanol
5	3.5905	0.403	2-Furanmethanol
6	3.848	1.0215	2-Furanmethanol
7	4.0368	1.9111	2-Cyclopenten-1-one, 2-methyl-
8	4.0826	6.2497	trans,trans-3,5-Heptadien-2-one
9	4.2543	0.3116	1-Penten-3-ol, 3-methyl-
10	4.3401	0.5976	Propanedioic acid, propyl-
11	4.4202	0.3097	1-Methylcycloheptene
12	4.5518	2.4842	Butyrolactone
13	4.7864	1.7081	2-Cyclopenten-1-one, 3-methyl-
14	5.0096	10.4877	Phenol
15	5.227	8.8624	Phenol
16	5.5474	1.3756	Butanal, 3-methyl-, oxime
17	5.6791	0.5016	Fampridine
18	5.8335	5.6333	1,2-Cyclopentanedione, 3-methyl-
19	5.9194	2.1904	Phenol, 2-methyl-
20	6.0853	1.575	Phenol, 2-methyl-
22	6.2055	1.9848	p-Cresol
23	6.3485	4.0248	Phenol, 3-methyl-
24	6.4229	2.8939	Phenol, 2‑methoxy-
25	6.5431	0.6614	3,3-Dimethylpiperidine
26	6.606	0.2693	1H-Imidazole,1-ethyl-2-methyl
27	6.6518	0.4287	2-Octen-1-ol, (E)-
28	6.7319	0.3314	Cyclooctyl isopropylphosphonofluoridate
29	6.7948	0.8872	Pyridine-D5-
30	6.8864	0.8311	Maltol
32	7.0237	0.3583	Furan, 4-methyl-2-propyl-
33	7.1897	1.2406	3-Buten-2-one, 4-(1-aziridinyl)-
34	7.2297	0.344	3-Methoxyhex-1-ene
35	7.2926	1.0535	Phenol, 2,4-dimethyl-
36	7.4128	0.6659	Phenol, 3-ethyl-
37	7.5158	2.7888	Phenol, 4-ethyl-
38	7.6703	0.3951	Phenol, 4-amino-3-methyl-
39	7.7332	0.3344	1,3-Dimethylimidazole-2(3H)-thione
40	7.8591	0.5247	Phenol, 3,5-dimethyl-
41	7.9106	0.5331	4,7,7-Trimethyl-5-(tetrahydropyran-2-yloxy)-bicyclo[2.2.1]heptan-2-one
42	7.985	1.15	1H-Pyrrole-2,5‑dione, 1-(hydroxymethyl)-
43	8.1509	7.0005	1,4:3,6-Dianhydro-.alpha.-d-glucopyranose
44	8.3569	0.2902	3,4,5,6,7,8-Hexahydro-2H-chromene
45	8.4027	0.492	3,4,5,6,7,8-Hexahydro-2H-chromene
48	8.8204	0.6899	1,2-Benzenediol, 4-methyl-
49	8.9234	0.3183	VII tropolone
50	8.9921	0.3254	Verbenol
51	9.1466	1.5095	Indolizine
52	9.3068	1.389	Hydroquinone
53	9.7989	0.4403	Phenol, 2,6-dimethoxy-
54	10.0049	0.9266	Orcinol
55	10.2624	0.3755	1H-Indole, 6-methyl-
56	10.846	0.8205	5-Ethylhydantoin
57	11.7558	0.8446	.beta.-d-Glucopyranose, 1,6-anhydro-
58	11.8073	0.8289	D-Allose
59	11.8759	0.9758	Pyrazine, 2,5-dimethyl-3-propyl-
60	12.2765	0.5954	3-Morpholino-1,2-propanediol
61	13.0776	0.2633	Acetate, (2-(3‑hydroxy-3-methyl-2-oxotetrahydro-1H-1-pyrrolyl)ethyl] ester
62	13.8844	0.2709	2-Octene, 3,7-dimethyl-, (Z)-
63	14.1361	0.3264	Ethanone, 1-(2,2-dimethylcyclopentyl)-
65	15.1146	0.4027	Phenol, 3,5-dimethoxy-
66	16.0187	1.9462	n-Hexadecanoic acid
67	17.5407	0.5891	9-Octadecenoic acid, (E)-
69	27.7086	1.0325	Cyclotrisiloxane, hexamethyl-

### Characterization of plantain and yam peels, bio-oil, and biochar obtained from the different temperature ranges

For the proximate analysis, the Kjeldahl method was used to determine the protein content [7], the Association of Official Analytical Chemists method [8] was used to determine the carbohydrate using Eq. (4).

(4) %CHO=100−%(protein+fat+ash+crudefibre+moisture)

X-Ray fluorescence (XRF) was used for the compositional analysis of the biochar, while the Gas Chromatography-Mass Spectroscopy (GC–MS) was used for the biochemical analysis of the obtained bio-oils.

## Declaration of Competing Interest

The authors declare that they have no known competing financial interests or personal relationships which have, or could be perceived to have, influenced the work reported in this article

## Appendix Supplementary materials

Image, application 1

Image, application 1
