# Supplementary material for: Experimental dataset investigating the effect of temperature in the presence or absence of catalysts on the pyrolysis of plantain and yam peels for bio-oil production
Source: Data Brief. 2020 Jun 3;31:105804. doi: 10.1016/j.dib.2020.105804 (PMC7300137; doi:10.1016/j.dib.2020.105804)
Supplement: Supplementary file 2 [file mmc2.docx]

**
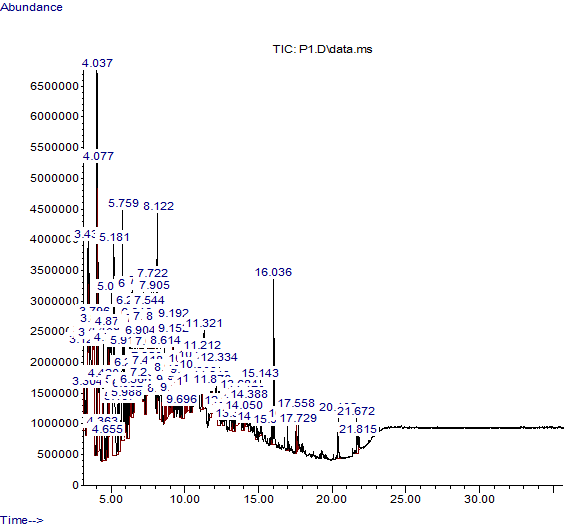
**

**Figure 1: GC-MS Chromatogram of the bio-oil obtained from the pyrolysis of plantain peel, in the absence a catalyst and at the temperature range of 250-350 ^o^C.**

**
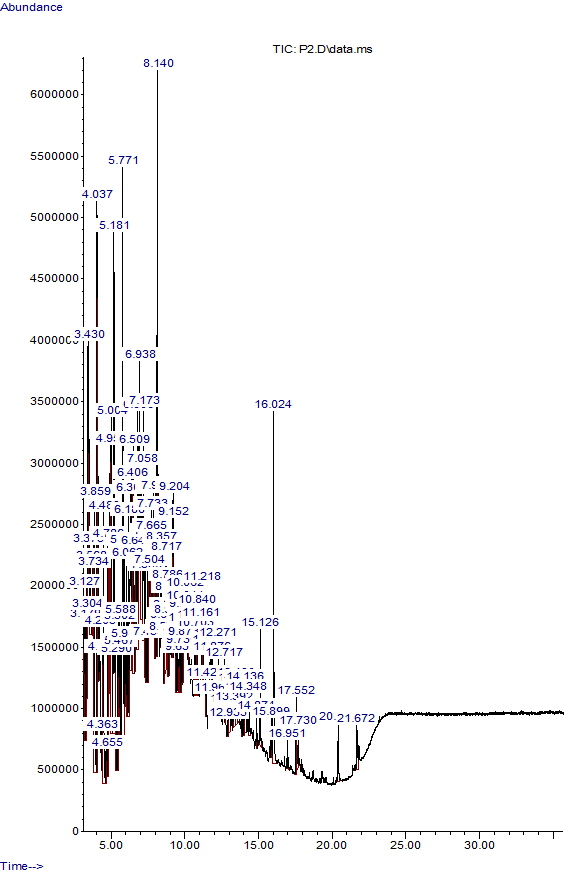
**

**Figure 2: GC-MS Chromatogram of the bio-oil obtained from the pyrolysis of plantain peel, in the absence a catalyst and at the temperature range of 350-450 ^o^C.**

**
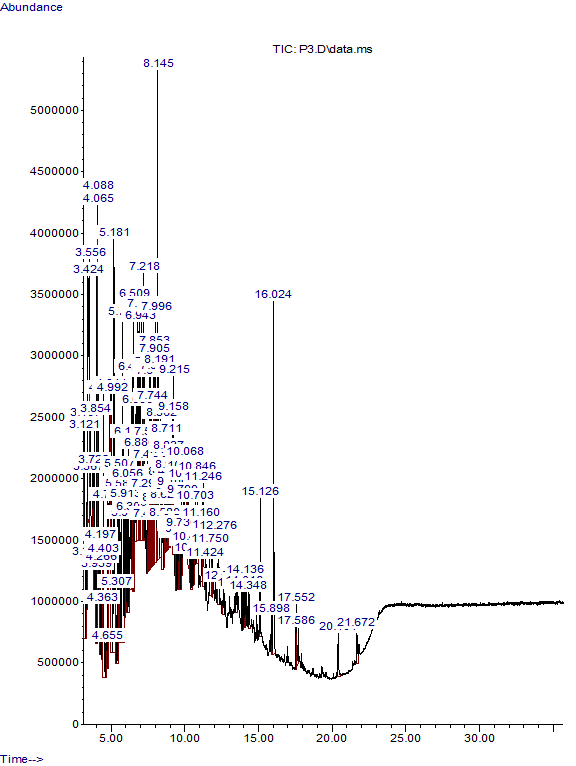
**

**Figure 3: GC-MS Chromatogram of the bio-oil obtained from the pyrolysis of plantain peel, in the absence a catalyst and at the temperature range of 450-550 ^o^C.**

**
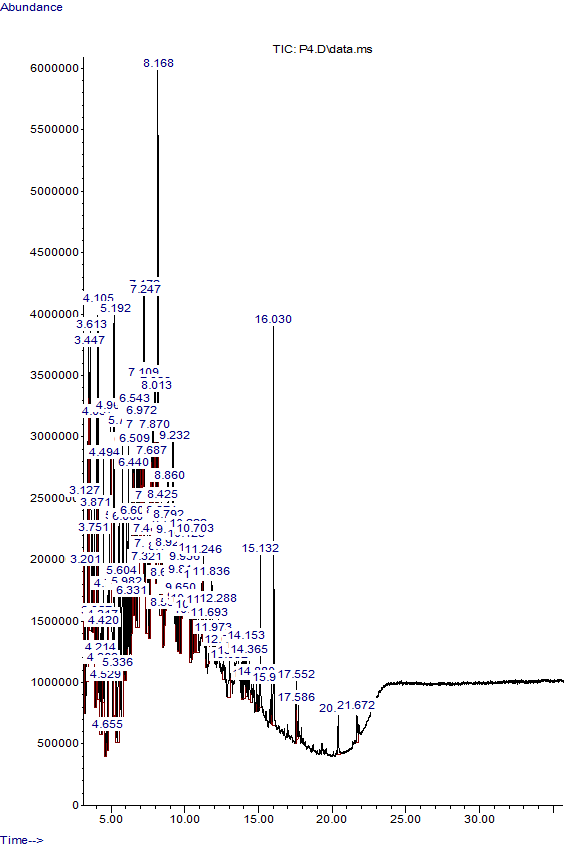
**

**Figure 4: GC-MS Chromatogram of the bio-oil obtained from the pyrolysis of plantain peel, in the absence a catalyst and at the temperature range of 550-650 ^o^C.**

**
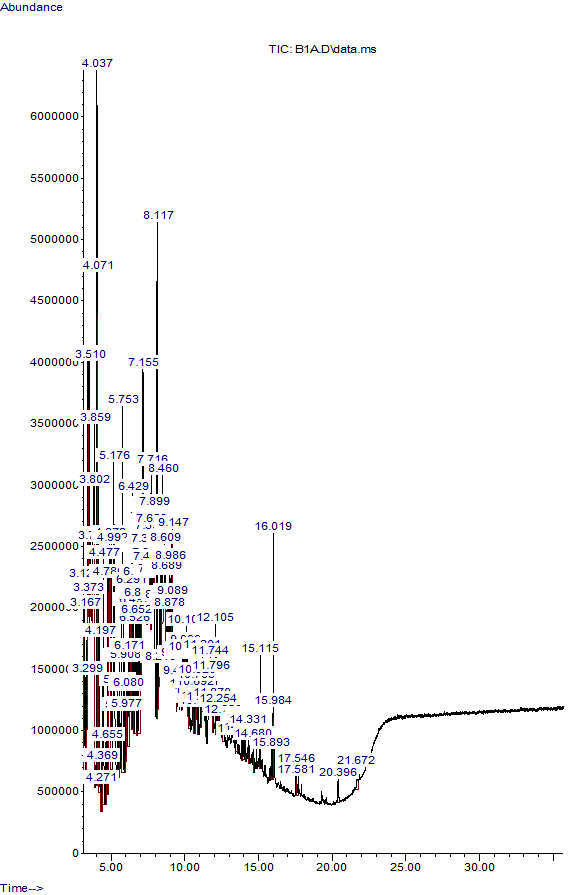
**

**Figure 5: GC-MS Chromatogram of the bio-oil obtained from the pyrolysis of plantain peel, using heterogeneous catalysis and at the temperature range of 250-350 ^o^C.**

**
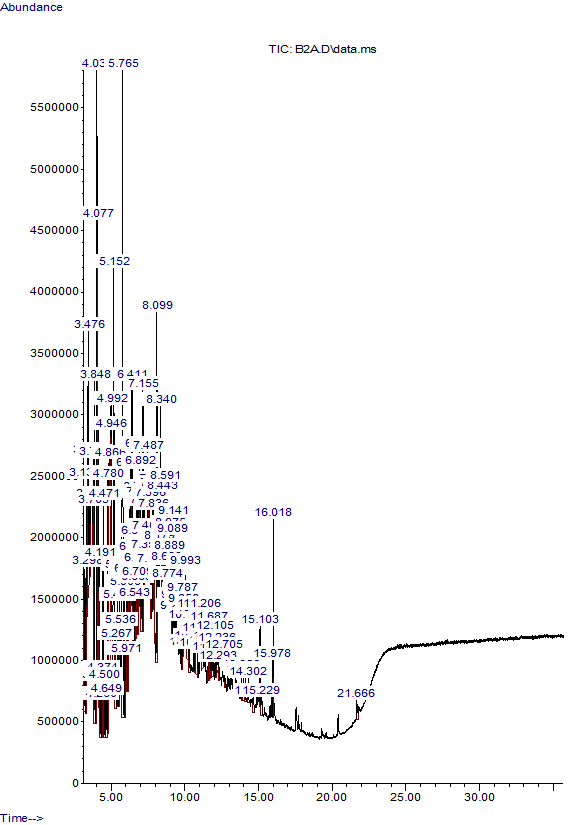
 Figure 6: GC-MS Chromatogram of the bio-oil obtained from the pyrolysis of plantain peel, using heterogeneous catalysis and at the temperature range of 350-450 ^o^C.**

**
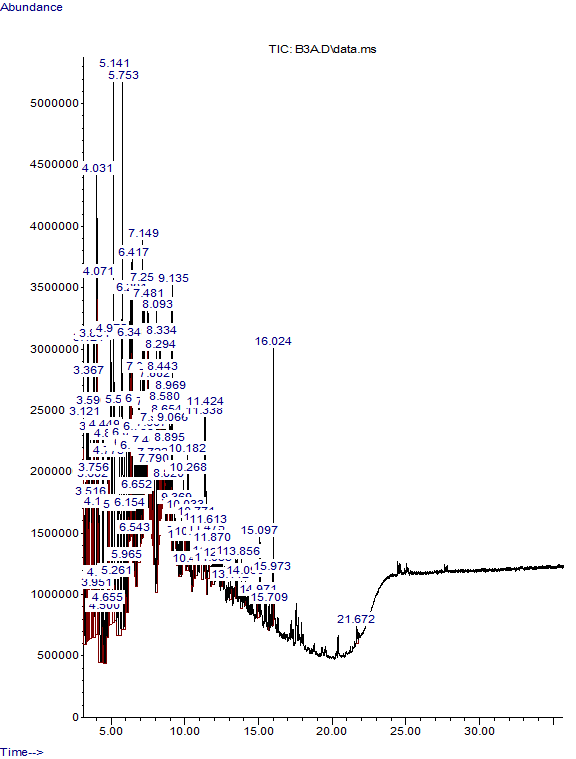
**

**Figure 7: GC-MS Chromatogram of the bio-oil obtained from the pyrolysis of plantain peel, using heterogeneous catalysis and at the temperature range of 450-550 ^o^C.**

**
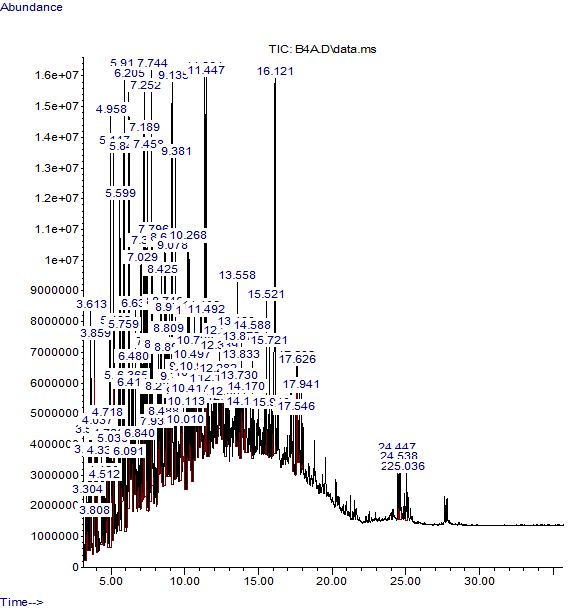
**

**Figure 8: GC-MS Chromatogram of the bio-oil obtained from the pyrolysis of plantain peel, using heterogeneous catalysis and at the temperature range of 550-650 ^o^C.**

**.**

**
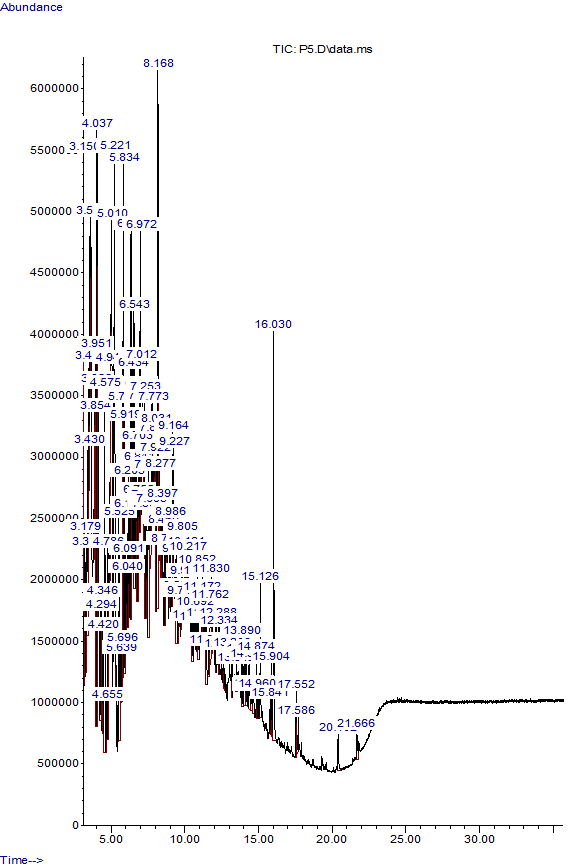
**

**Figure 9: GC-MS Chromatogram of the bio-oil obtained from the pyrolysis of plantain peel, using homogenous catalysis and at the temperature range of 200-300 ^o^C.**

**
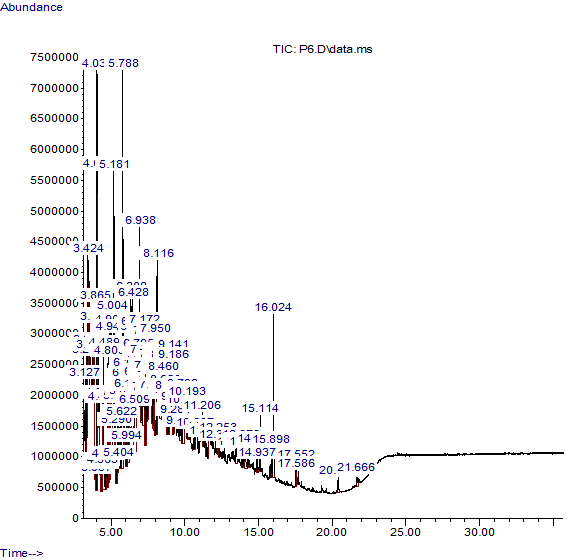
**

**Figure 10: GC-MS Chromatogram of the bio-oil obtained from the pyrolysis of plantain peel, using homogenous catalysis and at the temperature range of 300-400 ^o^C.**

**
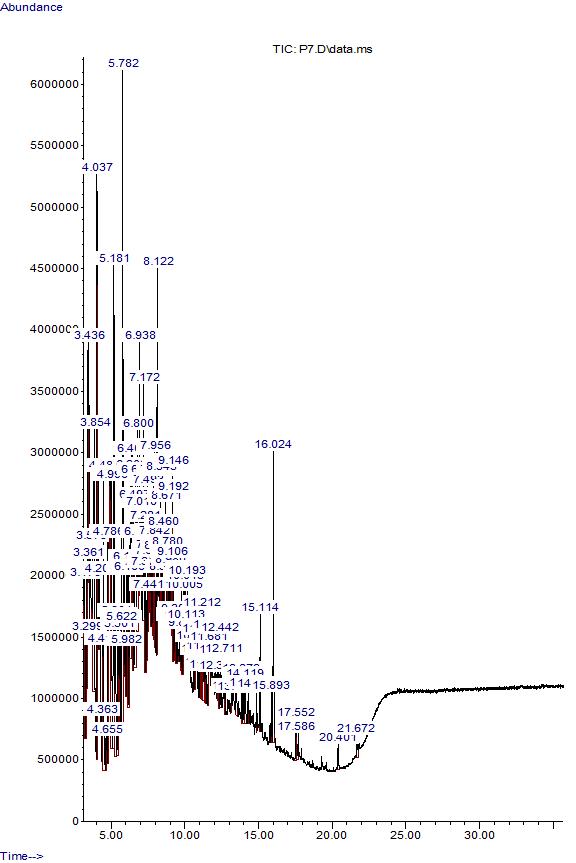
**

**Figure 11: GC-MS Chromatogram of the bio-oil obtained from the pyrolysis of plantain peel, using homogenous catalysis and at the temperature range of 400-500 ^o^C.**

**
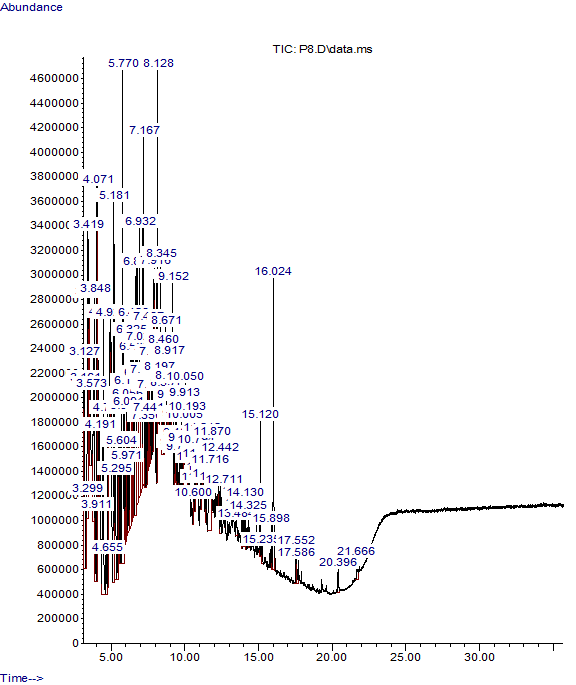
**

**Figure 12: GC-MS Chromatogram of the bio-oil obtained from the pyrolysis of plantain peel, using homogenous catalysis and at the temperature range of 500-600 ^o^C**

**
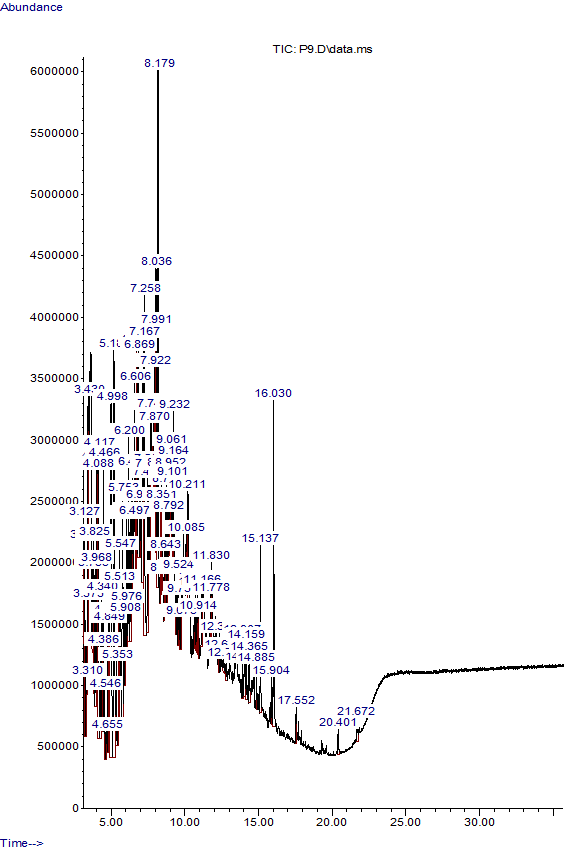
**

**Figure 13: GC-MS Chromatogram of the bio-oil obtained from the pyrolysis of plantain peel, using homogenous catalysis and at the temperature range of 600-500 ^o^C**

**
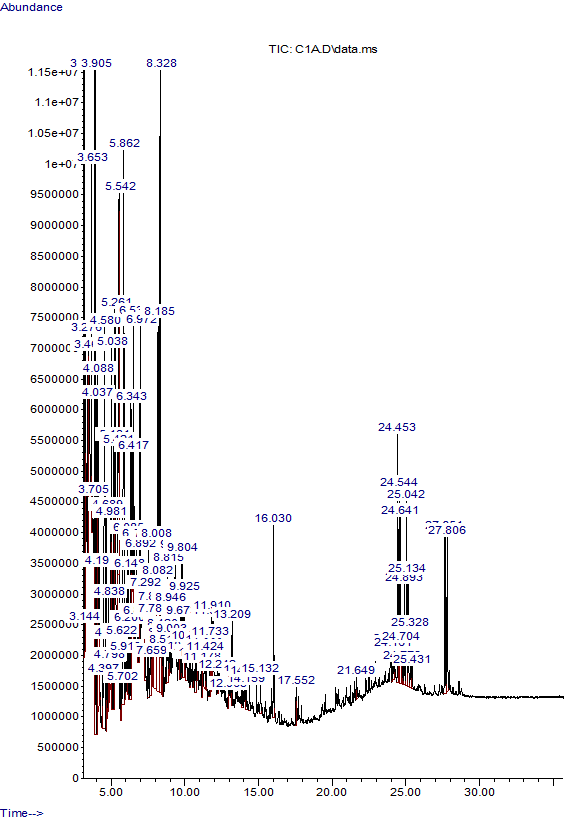
**

**Figure 14: GC-MS Chromatogram of the bio-oil obtained from the pyrolysis of yam peel, in the absence of a catalyst, and at the temperature range of 250-350 ^o^C**

**
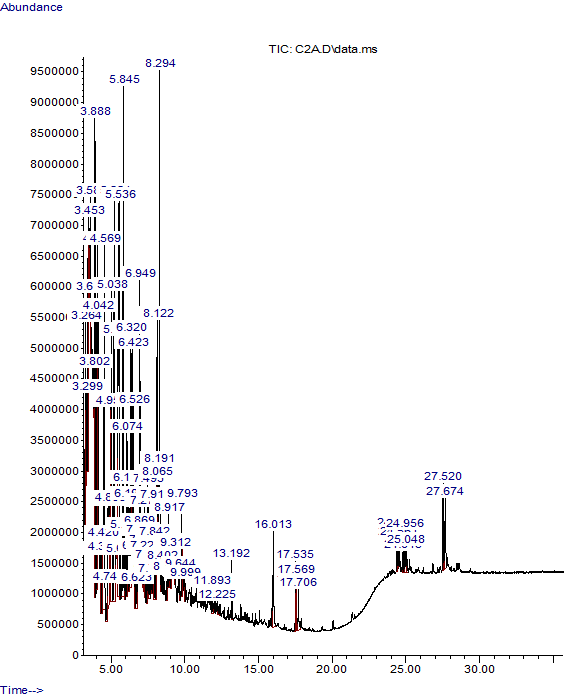
**

**Figure 15: GC-MS Chromatogram of the bio-oil obtained from the pyrolysis of yam peel, in the absence of a catalyst, and at the temperature range of 350-450 ^o^C**

**
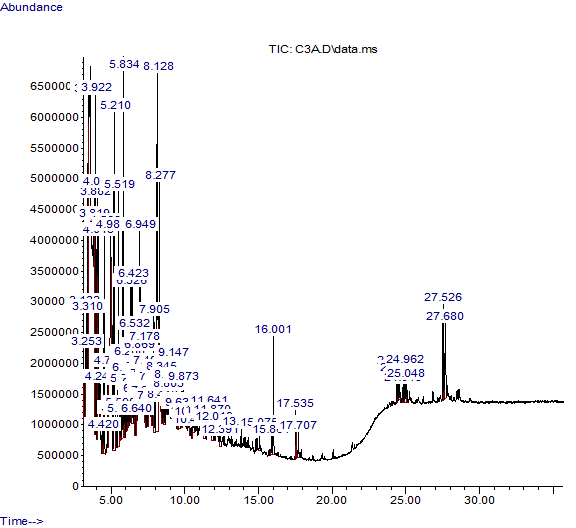
**

**Figure 16: GC-MS Chromatogram of the bio-oil obtained from the pyrolysis of yam peel, in the absence of a catalyst, and at the temperature range of 450-550 ^o^C**

**
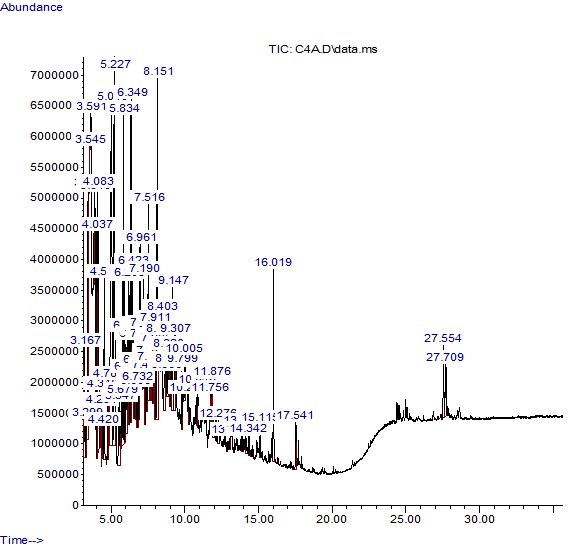
**

**Figure 17: GC-MS Chromatogram of the bio-oil obtained from the pyrolysis of yam peel, in the absence of a catalyst, and at the temperature range of 550-650 ^o^C**

**
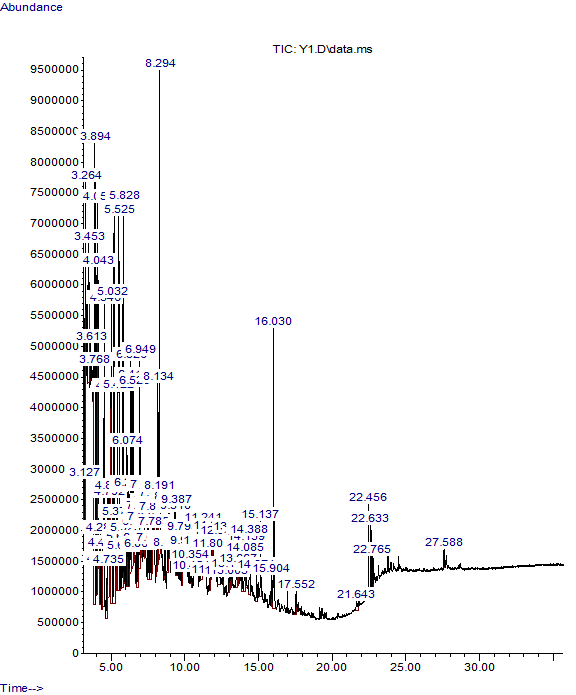
**

**Figure 18: GC-MS Chromatogram of the bio-oil obtained from the pyrolysis of yam peel, using homogenous catalysis, and at the temperature range of 200-300 ^o^C**
